# Nutraceuticals and Supplements in Management of Prediabetes and Diabetes

**DOI:** 10.3390/nu17010014

**Published:** 2024-12-24

**Authors:** Giuseppe Derosa, Angela D’Angelo, Fabrizio Angelini, Luca Belli, Arrigo F. G. Cicero, Roberto Da Ros, Giovanni De Pergola, Giovanni V. Gaudio, Alessandro Lupi, Giovanni Sartore, Federico A. Vignati, Pamela Maffioli

**Affiliations:** 1 SINut—Società Italiana di Nutraceutica, Via Guelfa, 9, 40138 Bologna, Italy; labmedmol@smatteo.pv.it (A.D.); afgcicero@gmail.com (A.F.G.C.); p.maffioli@smatteo.pv.it (P.M.); 2 CFC—Collegio Federativo di Cardiologia, Via Paolo Maspero, 5, 21100 Varese, Italy; gvgaudio@gmail.com (G.V.G.); lupialessandro1@gmail.com (A.L.); 3 Department of Internal Medicine and Therapeutics, University of Pavia, Via Aselli, 43/45, 27100 Pavia, Italy; 4 SINseB—Società Italiana Nutrizione, Sport e Benessere, Via Morimondo 26, 20143 Milano, Italy; fabrizio.angelini@gmail.com (F.A.); lucabelli.mc@gmail.com (L.B.); 5 AMD—Associazione Medici Diabetologi, Viale delle Milizie, 96, 00192 Roma, Italy; robertodaros73@gmail.com (R.D.R.); g.sartore@unipd.it (G.S.); 6 SIO—Società Italiana Obesità, Corso Italia, 115, 56125 Pisa, Italy; giovanni.depergola@irccsdebellis.it (G.D.P.); f.vignati@fastwebnet.it (F.A.V.)

**Keywords:** Dysglycemia, nutraceuticals, supplements, prediabetes, diabetes

## Abstract

Dysglycemia is a condition preceding diabetes mellitus. The two situations inherent in this condition are called impaired fasting glucose (IFG) and impaired glucose tolerance (IGT). If one of these situations is found in the patient, after the advice of an appropriate diet and physical activity, the addition of nutraceuticals or supplements can be considered, which can stop or delay the progression to diabetes mellitus over time. The purpose was to compile a systematic review about the use of nutraceuticals for treating diabetes and prediabetes and to offer a valuable resource for colleagues working on this crucial subject, thereby improving patient health. The added value of the paper compared to other reviews is that it was written by experts appointed by five different scientific societies dealing with diabetes, nutrition, and complications.

## 1. Introduction

Dysglycemia is a condition preceding the disease diabetes mellitus. The two situations inherent in this condition are called impaired fasting glucose (IFG) and impaired glucose tolerance (IGT), and it is possible to diagnose them through the loading curve with 75 g of glucose (OGTT). If one of these situations is found in the patient, after the advice of an appropriate diet and physical activity (at least 150–300 min of moderate-intensity aerobic physical activity, weekly) [1], the addition of nutraceuticals or supplements can be considered, which can stop or delay the progression to diabetes mellitus over time [2,3].

Dysglycemia is defined as having a fasting plasma glucose (FPG) ≥ 100 mg/dL or higher, according to the American Diabetes Association in 2021 [4], while type 2 diabetes mellitus (T2DM) is diagnosed by having two fasting plasma glucose readings of 126 mg/dL or higher, or by an occasional glucose level ≥ 200 mg/dL, along with symptoms of hyperglycemia in the preceding months.

When the FPG levels are ≥100 mg/dL and <126 mg/dL, and there is a concern of T2DM, an OGTT should be carried out to distinguish between IFG, with levels at the end of the second hour after glucose load less than 140 mg/dL; IGT, defined as two hours after test values in a range from 140 to 199 mg/dL; and diabetes, which is defined as blood sugar levels equal to or greater than 200 mg/dL.

The purpose was to compile a systematic review about the use of nutraceuticals for treating diabetes and prediabetes and to offer a valuable resource for colleagues working on this crucial subject, thereby improving patient health. The added value of the paper compared to other reviews is that it was written by experts appointed by five different scientific societies dealing with diabetes, nutrition, and complications.

## 2. Material and Methods

A systematic search strategy was developed to identify randomized controlled trials in both MEDLINE (National Library of Medicine, Bethesda, MD; 1996 through June 2023) and the Cochrane Register of Controlled Trials (The Cochrane Collaboration, Oxford, United Kingdom). The terms “nutraceutical”, “botanicals”, “dysglycemia”, “ascophyllum nodosum”, “fucus vesiculosus”, “banaba”, “berberine”, “cinnamomum”, “gymnemic acid”, “ilex paraguariensis”, “momordica charantia”, “morus alba”, “olea europaea”, alpha-lipoic acid, “omega-3 polyunsaturated fatty acids”, and “essential amino acids” were integrated into a randomized controlled trial-specific electronic search technique that used the Dickersin filter [5]. With the current review, we attempted to address some of the recent key research questions about the use of nutraceuticals in type 2 diabetes prevention and treatment: How might they work in conjunction with lifestyle changes? Which nutraceuticals are the most well-known for treating type 2 diabetes and prediabetes? What is the best evidence regarding their impacts and possible functions in glycemic control? What part might they play in regulating metabolism, reducing blood sugar, and enhancing insulin sensitivity?

## 3. Nutraceuticals

### 3.1. Ascophyllum nodosum (L.) and Fucus vesiculosus (L.)

*Ascophyllum nodosum* and *Fucus vesiculosus* are edible marine brown seaweeds that have been consumed by coastal populations in Asia, Britain, and other nations since ancient times [6].

Due to the presence of many bioactive chemicals, such as polyphenolics, phlorotannins, and fucoidans [7,8], the efficacy of these nutraceuticals in treating metabolic illnesses like obesity and type 2 diabetes has recently been established [9,10].

#### 3.1.1. Mechanisms of Action

**Figure 1:**
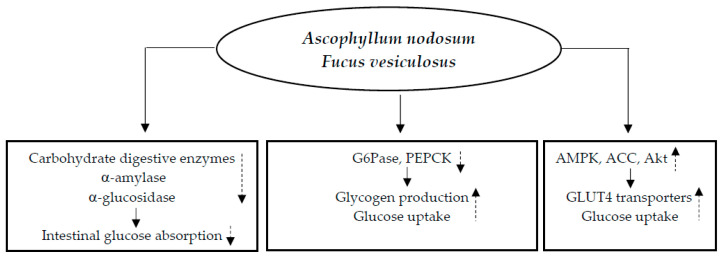
Mechanisms of action of *Ascophyllum nodosum* and *Fucus vesiculosus* hypoglycemic activity. ACC: acetyl-CoA carboxylase; Akt: serine/threonine kinase; AMPK: adenosine monophosphate-activated protein kinase; G6Pase: glucose-6-phosphatase; GLUT: glucose transporter; PEPCK: phosphoenolpyruvate carboxykinase. 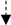: decrease; 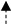: increase.

In vitro studies reported the potential hypoglycemic molecular mechanisms of bioactive compounds present in *Ascophyllum nodosum* and *Fucus vesiculosus*. These molecules have the ability to lower blood sugar levels via (1) inhibition of carbohydrate digestive enzymes, α-amylase, and α-glucosidase, thus delaying and decreasing intestinal absorption of glucose; (2) inhibition of hepatic enzymes [glucose-6-phosphatase (G6Pase) and phosphoenolpyruvate carboxykinase (PEPCK)], promoting glycogen production and glucose uptake at cellular level; and (3) upregulation of adenosine monophosphate-activated protein kinase (AMPK), acetyl-CoA carboxylase (ACC), and serine/threonine kinase (AKT), resulting in an increase in the quantity of glucose transporter 4 (GLUT4) present on the cell membrane and improving glucose uptake at the cellular level [11] (Figure 1).

#### 3.1.2. Clinical Trials

Some human studies investigated the hypoglycemic effects of *Ascophyllum nodosum* and *Fucus vesiculosus* (Table 1).

One trial evaluated the effect of *Ascophyllum nodosum* on post-prandial plasma glucose (PPG) in 12 men who were overweight or obese and consumed breakfast consisting of 100 g of bread plus 4% supplement. Ascophyllum nodosum was found not to have an impact on PPG levels during a test meal after four hours [12].

Another study showed that, in 43 healthy subjects, the use of two capsules per day each containing dried *Ascophyllum nodosum* 900 mg and 175 μg iodine for 6 weeks had no effect on FPG, fasting plasma insulin (FPI), and homeostasis model assessment index (HOMA index) in comparison to placebo [13].

Murray et al. investigated the effects of a powdered extract from Fucus vesiculosus on PPG and post-prandial plasma insulin (PPI) in a group of 38 healthy people. Before a carbohydrate load, the patients were given either 2 g of placebo or 500 mg and 2 g of the nutraceutical, which contained 140 mg and 560 mg of polyphenols, respectively. Two hours after consuming carbohydrates, no Fucus vesiculosus dosage changed PPG and PPI levels compared to placebo. Nonetheless, Asian participants were found to have a considerably greater insulin incremental area under the curve (iAUC) compared to non-Asian participants (*p* = 0.016), indicating that ethnicity has an impact on this parameter [14].

The same authors examined the effects of Fucus vesiculosus in the form of a powdered extract on PPG and PPI in the evening in 18 subjects with normal blood pressure (BP) and who had taken 2 g of the supplement, which included 1340 mg of fucoidan and 560 mg of polyphenols, or a placebo 30 min prior to a carbohydrate-rich meal. Three hours following supplement consumption, PPG and PPI showed no change in comparison to the placebo. *Fucus vesiculosus*, however, decreased females’ peak PPG (*p* = 0.018) compared to placebo, indicating that sex had an impact on this result. Furthermore, Asian populations had higher PPI iAUC and peak plasma insulin concentrations than non-Asian individuals (*p* = 0.07 for both), indicating the influence of ethnicity [11].

Several studies assessed the ability of *Fucus vesiculosus* and *Ascophyllum nodosum* alone and in combination with other nutraceuticals to reduce blood glucose levels.

In a 2011 study by Paradis et al., 23 healthy participants were enrolled and given a 500 mg combination of *Ascophyllum nodosum* and *Fucus vesiculosus* or placebo half an hour before consuming 50 g of carbohydrates from bread. Three hours after the test meal was consumed, the supplement was found to have no effect on the glucose response but to significantly reduce insulin iAUC (*p* = 0.04) and increase the insulin sensitivity index of Cederholm and Wibell (*p* < 0.05) in comparison to the placebo [15].

In a study conducted by De Martin et al., 50 patients who were overweight or obese were given a phytocomplex at the dose of three capsules daily, each containing 237.5 mg of Ascophyllum nodosum, 12.5 mg of Fucus vesiculosus, and 7.5 µg of chromium picolinate. The patients were followed for six months to assess the hypoglycemic effect of the combination. Fasting plasma glucose, FPI, and HOMA index were significantly reduced after 90 days (*p* < 0.05 for all) and 180 days of supplementation (*p* < 0.001 for FPG; *p* < 0.05 for FPI; *p* < 0.01 for HOMA index) compared to initial values [16].

Another trial examined how this combination of nutraceuticals affected the glycemic status of 65 dysglycemic patients who were randomly assigned to receive a placebo or three capsules of supplement daily for six months. The nutraceutical agent significantly reduced FPG, PPG, HOMA index, and glycated hemoglobin (HbA_1c_) (*p* < 0.05 for all) at the end of therapy as compared to placebo [17].

In a further trial with the same design as the one mentioned above and 175 T2DM patients participating, the same authors assessed the effectiveness of the nutraceutical combination on glucose indices. The supplement significantly decreased FPG, PPG, and HbA_1c_ compared to baseline (*p* < 0.01 for FPG and PPG; *p* < 0.05 for HbA_1c_) and to placebo (*p* < 0.05 for all) [18].

### 3.2. Banaba [(Lagerstroemia speciosa (L.) Pers]

Banaba [*Lagerstroemia speciosa* (L.) Pers] is a plant found in tropical regions, including the Philippines, India, Malaysia, Southern China, and Australia. These countries use the plant as a traditional medicine for its supposed health benefits. Diabetes and kidney disease are treated in the Philippines with a tea made from the leaves. The hypoglycemic effect of banaba is linked to the abundant presence of ellagitannins and corosolic acid in its leaves [19,20].

#### 3.2.1. Mechanisms of Action

**Figure 2:**
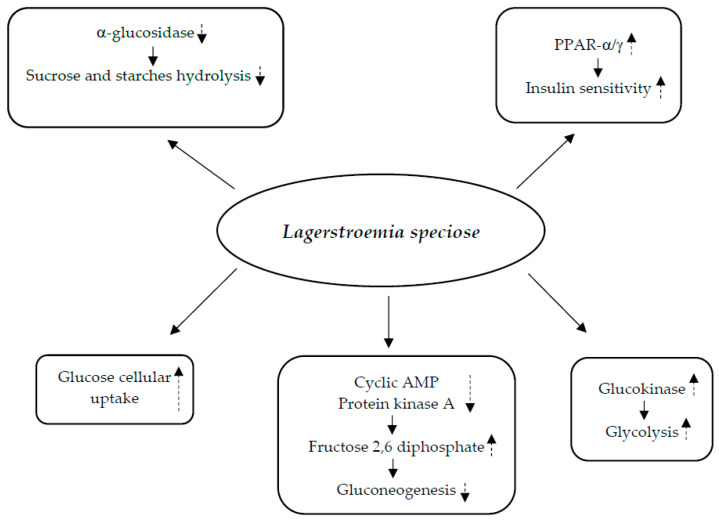
Hypoglycemic mechanisms of action of *Lagerstroemia speciose*. PPAR-α/γ: peroxisome proliferator-activated receptor-α/γ. 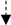: decrease; 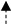: increase.

As reported in studies in vivo and in vitro, the hypoglycemic effect of banaba appears to be due to different mechanisms: (1) improvement in the cellular absorption of glucose; (2) decrease in the hydrolysis of starches and sucrose; (3) inhibition of protein kinase A activity and reduction in cyclic AMP lead to an increase in fructose 2,6 diphosphate levels that in turn decreases gluconeogenesis; (4) boosting glucokinase activity to promote glycolysis; (5) improvement of insulin sensitivity by increased expression of adipose tissue *peroxisome proliferator-activated receptor*-γ (PPAR-γ) mRNA and liver peroxisome proliferator-activated receptor-α (PPAR-α) mRNA; and (6) inhibition of α-glucosidase activity [20] (Figure 2).

#### 3.2.2. Clinical Trials

The human studies that analyzed the hypoglycemic effect of banaba and its components are reported in Table 2.

Fifteen subjects with IFG were treated with 100 mg/day of a water-soluble banaba extract in the pill formulation. The supplement reduced FPG levels (16.60%). Since corosolic acid has poor aqueous solubility, ellagitannins are mostly responsible for the hypoglycemic impact, even though the extract was not standardized, and its constituents were not identified [21].

Judy et al. examined the effect of a banaba extract standardized to 1% corosolic acid on decreasing blood glucose levels in ten patients with T2DM. Over the course of two weeks, three doses of the supplement (16, 32, and 48 mg) in either the soft-gel or hard-gel formulation were given. The highest dosage of nutraceuticals resulted in a significant reduction in FPG in both formulations at the conclusion of therapy (*p* ≤ 0.002 for soft-gel form; *p* ≤ 0.001 for hard-gel form). Compared to the dry-powder formulation, the soft-gel ones have greater bioavailability since the decrease was highest in that formulation. Nevertheless, it is unknown if tannins and corosolic acid work alone or together to reduce FPG [19].

In another study, 12 participants with IFG were given a daily soft-gel capsule of banaba extract containing 10 mg of corosolic acid. Fasting plasma glucose and 1h-PPG levels have been shown to drop by 12% after two weeks of administration. The investigation has not made it clear whether corosolic acid alone or in conjunction with tannins is responsible for the hypoglycemic action [22].

Five minutes prior to the OGTT, 31 participants ingested a capsule containing either a placebo or 10 mg of corosolic acid on separate occasions. There was a seven-day gap between treatments. It was observed that the supplement reduced plasma glucose levels from 60 min to 120 min, and this decrease was statistically significant at 90 min (*p* < 0.05) compared to placebo. Furthermore, the use of 99% pure corosolic acid is undoubtedly responsible for the hypoglycemic effect [23].

Choi et al. assessed the hypoglycemic effect of 300 mg/day banaba extract (BE; 0.3% corosolic acid), 2 g/day soybean leaf extract (SLE), or 2 g/day placebo during meals for 12 weeks in 45 prediabetics. Both supplements significantly decreased HbA_1c_ levels in a similar way (*p* < 0.05 for BE and SLE), and they also showed lower baseline-adjusted final FPG levels (*p* < 0.05 for BE and SLE) and HOMA-IR (*p* < 0.05 for BE and SLE) compared to placebo [24].

Some studies indicated that banaba has hypoglycemic properties when combined with other nutraceuticals.

In one study, the effects of a tablet product including an aqueous banaba extract, green tea, green coffee, and Garcinia were documented. The product was given to 24 mildly diabetic participants three times a day, at a dose of three tablets. An average decrease of 13.50% in FPG levels was noted. Nevertheless, the elements accountable for the hypoglycemic impact remained unidentified [25].

Another trial found no significant differences in FPG, FPI, HOMA-IR, or HbA_1c_ levels between the baseline and the placebo after treating 62 IGT and mild T2DM patients with a 6 g/day combination of banaba leaf water extract, mulberry leaf water extract, and Korean red ginseng powder in equal ratios for six months. Nonetheless, during OGTT, the treatment group showed a substantial decrease in glucose AUC (*p* < 0.05) and a tendency for insulin AUC to lower, which did not achieve statistical significance compared to baseline [26].

In an 8-week trial, 40 IFG patients in primary prevention of cardiovascular disease took 250 mg of Lagerstroemia speciosa extract combined with 155 mg of berberine, 125 mg of curcumin extract, 110 mg of α-lipoic acid, 1.3 μg of chromium picolinate, and 0.15 mg of folic acid, or placebo, as pills twice a day. Patients administered the nutraceutical combination showed a significant reduction in FPG levels (*p* < 0.05) compared to baseline, as did the placebo group (*p* < 0.05), whose reduction was, however, lower than that of the group taking the supplement. Furthermore, FPI and HOMA-IR were considerably reduced by the nutraceutical combination in comparison to both baseline (*p* < 0.05 for both) and placebo (*p* < 0.05 for both). Nevertheless, more medium- and long-term studies with a larger sample size are needed to corroborate these results [27].

Banaba’s effects in association with berberine, curcumin, inositol, and chromium picolinate have recently been demonstrated in patients with IFG or IGT who are not receiving any hypoglycemic agent. For three months, a total of 148 participants were given one pill each day that included 200 mg of berberine, 200 mg of curcumin, 300 mg of inositol, 40 mg of banaba, and 100 mg of chromium picolinate, or a placebo. The nutraceutical combination decreased FPG, PPG, and HbA_1c_ levels at the end of treatment compared to baseline (*p* < 0.05 for all) and to placebo (*p* < 0.05 for all). There was also a reduction in HOMA-IR and an increase in FPI in comparison to baseline (*p* < 0.05 for both) and to placebo (*p* < 0.05 for both) after supplementation. It is likely that the combination of berberine and the other nutraceuticals improved the glycemic indices observed in this study [28].

### 3.3. Berberis

China’s traditional medicine uses berberine, an isoquinoline alkaloid, primarily to treat gastrointestinal tract illnesses. The hypoglycemic properties of berberine have led to its usage in the treatment of diabetes in more recent times. Numerous plant families are found to contain this alkaloid: Annonaceae, Berberidaceae, Menispermaceae, Papaveraceae, Ranunculaceae, and Rutaceae.

It is well recognized that the genus Berberis is the most widely distributed natural source of berberine among these therapeutic plants. The sections of plants that were determined to contain the highest concentration of berberine are the bark and roots, despite the fact that this compound is generally present in the stems, bark, and roots of the plants.

Furthermore, the highest concentration of berberine, between 5.2 and 7.7%, is found in Coptis rhizoma and barberry [29,30]. The most often utilized chemical form, according to numerous clinical studies [31,32,33,34,35] is berberine hydrochloride.

#### 3.3.1. Mechanisms of Action

**Figure 3:**
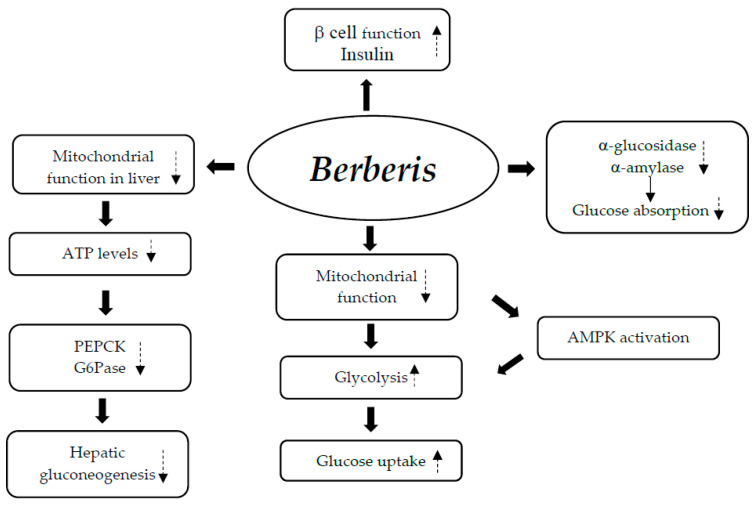
Hypoglycemic mechanisms of action of *Berberis*. AMPK: adenosine monophosphate-activated protein kinase; G6Pase: glucose-6-phosphatase; PEPCK: phosphoenolpyruvate carboxykinase. 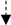: decrease; 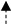: increase.

Berberine may regulate glucose metabolism through a variety of mechanisms that comprise (1) the restoration of depleted islets, which improves pancreatic β cell function and increases insulin sensitivity; (2) the inhibition of α-amylase and α-glucosidase activity, which lowers intestinal glucose absorption; (3) the induction of glycolysis, which stimulates glucose uptake and is brought on by the inhibition of mitochondrial function, resulting in the activation of the AMPK pathway; and (4) the suppression of hepatic gluconeogenesis as a result of a reduction in the expression of gluconeogenic genes (PEPCK and G6Pase). Adenosine triphosphate (ATP) depletion brought on by the liver’s inhibition of mitochondrial function is the cause of this decline (Figure 3) [30,36,37,38].

#### 3.3.2. Clinical Trials

Berberine was initially reported to lower blood glucose levels in 1988, when it was used to treat diarrhea in sixty T2DM patients who were not insulin-dependent [39]. Since then, berberine’s hypoglycemic effect has been the subject of other studies (Table 3).

Yin et al. evaluated the effects of giving 36 newly diagnosed T2DM participants 1.5 g/day of either metformin or berberine for three months. The supplement was found to significantly lower FPG, PPG, and HbA_1c_ (*p* < 0.01 for all) compared to initial values [33].

It has also been noted that berberine and metformin have comparable effects on blood glucose levels. In addition, the authors looked at the efficacy of berberine 1.5 g/day given to 48 diabetics with inadequately controlled glucose levels for three months, in addition to standard hypoglycemic drugs, such metformin, acarbose, sulfonylureas, or insulin. Berberine determined a significant lowering of FPG, PPG, and HbA_1c_ levels (*p* < 0.001 for all) in comparison to baseline. The supplement also decreased FPI levels (−9.90 µU/mL, −28.10%, *p* < 0.01) and HOMA-IR (−6.80, −44.70%, *p* < 0.001) compared to initial values. Berberine improved HbA_1c_ to a level comparable to metformin [40], as reported by Yin et al. [33].

In a cohort of 116 individuals with recent-onset T2DM with dyslipidemia, berberine consumption at a dose of 1 g/day for three months was found to reduce the concentrations of FPG, PPG, and HbA_1c_ compared to baseline (*p* < 0.0001 for all) and to placebo (*p* < 0.0001 for all). There was no significant difference in HOMA-IR, FPI, or PPI values between berberine and placebo [34]. In this trial, berberine reduced HbA_1c_ in a manner similar to that obtained with conventional oral hypoglycemic drugs [41].

Another study enrolled 97 T2DM patients who received 1 g/day of berberine, 1.5 g/day of metformin, or 4 mg/day of rosiglitazone for 2 months. It was shown that berberine caused a significant reduction in FPG and HbA_1c_ levels (*p* < 0.001 for both) compared to baseline. These parameters were lowered in a similar manner with metformin (*p* < 0.001 for both) and rosiglitazone administration (*p* < 0.01 for FPG; *p* < 0.001 for HbA_1c_). Furthermore, berberine treatment raised the proportion of peripheral blood cells expressing the insulin receptor (*p* < 0.01) and decreased insulin levels (*p* < 0.01).

The hypoglycemic action of berberine, consumed at a dose of 1 g/day for two months, was also observed in 35 subjects affected by chronic hepatitis B and hepatitis C with T2DM or IFG. The FPG levels in T2DM (*p* < 0.01 for the hepatitis B and C group) and IFG patients (*p* < 0.01 for the hepatitis B and C group) were decreased in comparison to baseline in both the hepatitis B and C groups after supplementation. Furthermore, after berberine therapy, these patients’ liver enzyme levels significantly decreased [35].

According to Gu et al., 60 individuals with dyslipidemia and recently diagnosed T2DM exhibited improvements in their glycemic parameters when they took 1 g/day of berberine for 3 months. It was found that FPG, PPG, and HbA_1c_ levels were decreased compared to initial values (*p* < 0.001 for all) and to placebo (*p* = 0.008 for FPG; *p* = 0.001 for PPG; *p* = 0.004 for HbA_1c_) following berberine consumption [42].

The hypoglycemic effect of berberine in combination with other nutraceuticals was shown in some clinical trials.

In the study by Di Pierro et al., 22 T2DM patients with poor glycemic control and treated with standard hypoglycemic agents received two tablets per day each containing 500 mg of berberine and 105 mg of Silybum marianum extract. After three months of treatment, the supplement significantly decreased HbA_1c_ (*p* = 0.003), FPI, and HOMA-IR (*p* = 0.04 for both) compared to initial values [43].

The effect of *Berberis aristata* with *Silybum marianum* compared to *Berberis aristata* alone was examined by the same authors in 69 patients with poorly controlled type 2 diabetes and undergoing diet and therapy with traditional oral hypoglycemic agents. Patients were administered 1 g/day of berberine alone or a combination of berberine, 1 g/day, and silymarin, 210 mg/day, as add-on therapy. After 4 months of supplementation, both treatments were determined to result in similar reductions in FPG (*p* = 0.006 for berberine plus silymarin; *p* = 0.007 for berberine alone) and HbA_1c_ (*p* < 0.001 for berberine plus silymarin and berberine alone) compared to initial values; however, the nutraceutical combination resulted in a greater decrease in HbA_1c_ levels (*p* < 0.05) compared to berberine alone [44].

A 14-month study enrolled 102 euglycemic dyslipidemic subjects that, at baseline, followed a low-calorie diet and performed physical activity for 6 months and then randomly assigned to receive an association of berberine, 1 g/day, and silymarin, 210 mg/day, or placebo for 3 months. They received the same supplement or a placebo for 3 more months following a 2-month break. During glucagon stimulation test, in the nutraceutical group, there was an increase in FPG and *C*-peptide after 6 min (*p* < 0.05 for both) versus time 0, while, in the placebo group, the levels of these markers at time 0 were greater than those at time 0 at baseline (*p* < 0.05), at randomization (*p* < 0.05), and at the end of study (*p* < 0.05). Additionally, in the placebo group, at 6 min from the glucagon injection, the supplement resulted in a lesser degree of increase in FPG and in a greater degree of increase in C-peptide at baseline (*p* < 0.05 for both), at randomization (*p* < 0.05 for both), and upon study completion (*p* < 0.05 for both) [45].

Another study included 105 euglycemic, overweight, dyslipidemic patients who took a combination of *Berberis aristata* and *Silybum marianum* according to the design previously mentioned [45]. The supplement decreased FPI levels and HOMA-IR, thus improving insulin resistance. More specifically, a significant decrease in both markers 3 months after randomization was observed compared to initial values (*p* < 0.05 for both) and to placebo (*p* < 0.05 for both). When the treatment was stopped, FPI and HOMA-IR levels increased (*p* < 0.05 for both) compared to 3 months following randomization, whereas, when supplementation was resumed, these parameters decreased once more compared to wash-out (*p* < 0.05 for both) and placebo (*p* < 0.05 for both) [46].

A sample of 137 euglycemic, dyslipidemic subjects who are unable to tolerate large doses of statins were given a combination of berberine, 1 g/day, and *Silybum marianum*, 210 mg/day, or placebo. After 6 months of supplementation, FPG, FPI, and HOMA-IR significantly decreased compared to initial values (*p* < 0.05 for all) and to placebo (*p* < 0.05 for all) [47].

*Berberis aristata* and *Silybum marianum* combination was also shown to improve the glycemic profile in 45 T2DM patients with hypercholesterolemia and statins intolerance who took the supplement (berberine, 1 g/day; and silymarin, 210 mg/day) alone or in combination with low-dose statins or ezetimibe. After 6 months, FPG levels were reduced by supplementation alone (*p* < 0.05), and after 12 months, by all treatments (*p* < 0.05 for all), compared to baseline. After 6 months, this parameter was significantly decreased both by the nutraceutical alone and plus statins (*p* < 0.05 for both) compared to the nutraceutical plus ezetimibe. All treatments reduced HbA_1c_ after 6 months (*p* < 0.05 for all) and at the end of treatment (*p* < 0.05 for nutraceutical alone and plus ezetimibe; *p* < 0.01 for nutraceutical plus statins) compared to initial values [48].

The effects of *Berberis aristata* plus *Silybum marianum* were also studied in 85 T1DM (type 1 diabetes mellitus) patients on insulin therapy for a minimum of three months who were also following a low-calorie diet and increasing physical activity. It was found that after 6 months of the consumption of the nutraceutical (1 g/day of berberine with 210 mg/day of silymarin) as additional treatment, FPG and PPG were reduced compared to baseline (*p* < 0.05 for both) and to placebo (*p* < 0.05 for both). In contrast, HbA_1c_ was only decreased when compared to levels prior to supplementation (*p* < 0.05). In addition, the nutraceutical reduced total amount of insulin used (*p* < 0.05 versus baseline and placebo) as well as insulin use at meals (*p* < 0.05 versus baseline and placebo for breakfast; *p* < 0.01 versus baseline and placebo; for lunch and for dinner) and at bedtime (*p* < 0.05 versus baseline and placebo) [49].

A retrospective study included 226 dyslipidemic patients naïve and intolerant to statins who were treated with a supplement, in tablet form, containing 500 mg of berberine from *Berberis aristata*, 105 mg of silymarin from *Silybum marianum*, and 10 mg of monacolins K and KA from *Monascus purpureus* fermented rice for 6 months. Both the patients who were given the supplement alone and those who took the supplement as an additional treatment exhibited a decrease in HbA_1c_ (*p* < 0.05 for supplement alone and as add-on therapy) and HOMA-IR (*p* < 0.05 for supplement alone and as add-on therapy) compared to initial values; however, the supplement alone reduced the levels of these parameters to a slightly greater extent than supplementation as an adjunctive therapy [50].

The supplement administered in the study described above was examined in another two trials [51,52]. The first reported that 143 subjects at low cardiovascular risk showed a significant reduction in FPG and HOMA-IR (*p* < 0.05 for both) compared to placebo, together with an increase in FPI levels compared both to baseline and placebo (*p* < 0.05 for both) after 3 months of supplementation [51]. The second trial was a retrospective analysis comprising 59 patients with T2DM and dyslipidemia under treatment with a low-calorie diet, physical activity, and oral hypoglycemic drugs. In the group of 31 patients treated with one tablet/day of nutraceutical combination for 6 months, the HbA_1c_ values were reduced (*p* < 0.05) compared to those before the supplementation [52].

Based on these findings, the authors conclude that the berberine present in the nutraceutical combination is probably responsible for its hypoglycemic effects.

A group of 64 patients with metabolic syndrome received 500 mg of berberine, 10 mg of policosanol, and red yeast rice (monacolin K 3 mg) in tablet form daily. A significant decrease was found in FPG (*p* = 0.006) compared to initial values and FPI (*p* = 0.042), together with mean PPI (*p* = 0.023), compared to placebo. The nutraceuticals combination also reduced HOMA-IR compared to baseline (*p* = 0.019) and to placebo (*p* = 0.023) [31].

In addition, the effects of two distinct nutraceutical combinations on glucose homeostasis were previously reported. These associations contain berberine, Lagerstroemia speciosa, curcumin, α-lipoic acid, chromium picolinate, folic acid, and berberine, banaba, curcumin, inositol, and chromium picolinate, respectively [27,28].

Moreover, in a randomized controlled trial in T2DM patients, a significant reduction in FPG and HbA_1c_ (*p* < 0.01 for both) was observed only in the group treated with a nutraceutical formulation based on berberine, hesperidin, and chromium picolinate, in addition to metformin, compared with the control group [53].

**Table 3:** Overview of clinical trials on glucose-lowering activity of *Berberis*.

First Author and Year	Participants	Therapy and Duration	Findings
Yin, J. (2008) [33]	(1) Patients with T2DM (n = 36) (2) Patients with poorly controlled T2DM (n = 48)	(1) 1.5 g/day berberine or metformin (2) 1.5 g/day berberine plus hypoglycemic agents 3 months	(1) FPG, PPG, and HbA_1c_ decrease (2) FPG, PPG, HbA_1c_, FPI, and HOMA-IR decrease
Zhang, Y. (2008) [34]	Patients with T2DM and dyslipidemia (n = 116)	1.0 g/day 3 months	FPG, PPG, and HbA_1c_ decrease
Zhang, H. (2010) [35]	(1) T2DM patients (n = 97) (2) T2DM or IFG patients with chronic hepatitis B and C (n = 35)	(1) 1.0 g/day berberine or 1.5 g/day metformin or 4 mg/day rosiglitazone (2) 1 g/day berberine 2 months	(1) FPG and HbA_1c_ decrease Insulin levels decrease Percentage of peripheral blood lymphocytes increase (2) FPG and liver enzymes decrease
Gu, Y. (2010) [42]	Patients with T2DM and dyslipidemia (n = 60)	1.0 g/day 3 months	FPG, PPG, and HbA_1c_ decrease
Di Pierro, F. (2012) [43]	T2DM patients (n = 22)	1 g/day berberine + 210 mg/day silymarin 3 months	HBA_1c_ and FPI decrease
Di Pierro, F. (2013) [44]	T2DM patients (n = 69)	1 g/day berberine + 210 mg/day silymarin or 1 g/day berberine 4 months	FPG and HbA_1c_ decrease
Derosa, G. (2013) [45]	Patients with dyslipidemia (n = 102)	1 g/day berberine + 210 mg/day silymarin 14 months	FPG and C-peptide after 6 min glucagon test increase FPG lower increase and C-peptide higher increase during glucagon test
Derosa, G. (2013) [46]	Patients with obesity and dyslipidemia (n = 105)	1 g/day berberine + 210 mg/day silymarin 14 months	FPI and HOMA-IR decrease
Derosa, G. (2015) [47]	Dyslipidemic patients (n = 137)	1 g/day berberine + 210 mg/day silymarin 6 months	FPG, FPI, and HOMA index decrease
Di Pierro, F. (2015) [48]	Patients with T2DM and hypercholesterolemia (n = 45)	1 g/day berberine + 210 mg/day silymarin or 1 g/day berberine + 210 mg/day silymarin with low dose statins or 1 g/day berberine + 210 mg/day silymarin with ezetimibe 12 months	FPG and HbA_1c_ decrease
Derosa, G. (2016) [49]	Type 1 diabetes mellitus patients (n = 85)	1 g/day berberine + 210 mg/day silymarin 6 months	FPG, PPG, and HbA_1c_ decrease Insulin consumption decrease
Di Pierro, F. (2016) [50]	Dyslipidemic patients (n = 226)	500 mg/day berberine + 105 mg/day silymarin + 10 mg/day monacolins K and KA 6 months	HbA_1c_ and HOMA-IR decrease
Derosa, G. (2017) [51]	Low-cardiovascular-risk patients (n = 143)	500 mg/day berberine + 105 mg/day silymarin + 10 mg/day monacolins K and KA 3 months	FPG and HOMA index decrease FPI increase
Di Pierro, F. (2018) [52]	Patients with T2DM and dyslipidemia (n = 59)	500 mg/day berberine + 105 mg/day silymarin + 10 mg/day monacolins K and KA 6 months	HbA_1c_ decrease
Affuso, F. (2012) [31]	Patients with metabolic syndrome (n = 64)	500 mg/day berberine + 10 mg/day policosanol + 3 mg/day monacolin K 18 weeks	FPG, FPI, PPI, and HOMA-IR decrease
Cicero, A.F.G. (2017) [27]	IFG patients (n = 40)	310 mg/day berberine + 500 mg/day *Lagerstroemia speciosa* + 250 mg/day curcumin + 2.6 μg/day chromium picolinate + 0.30 mg/day folic acid + 220 mg/day α-lipoic acid 8 weeks	FPG, FPI, and HOMA index decrease
Derosa, G. (2020) [28]	IFG and IGT patients (n = 148)	200 mg/day berberine + 200 mg/day curcumin + 300 mg/day inositol + 40 mg/day banaba + 100 μg/day chromium picolinate 3 months	FPG, PPG, HbA_1c_, and HOMA-IR decrease FPI increase
Sartore, G. (2021) [53]	T2DM (n = 20)	250 mg/day berberine + 200 mg/day hesperidin + 200 μg/day chromium picolinate 3 months	FPG and HbA_1c_ decrease

FPG: fasting plasma glucose; FPI: fasting plasma insulin; HbA_1c_: glycated hemoglobin; HOMA-IR: homeostatic model assessment of insulin resistance; IFG: impaired fasting glucose; IGT: impaired glucose tolerance; PPG: post-prandial plasma glucose; PPI: post-prandial plasma insulin; T2DM: type 2 diabetes mellitus.

### 3.4. Cinnamomum

The genus *Cinnamomum* belong to the Lauraceae family and includes *Cinnamomum zeylanicum* or *Cinnamomum verum*, also called Ceylon Cinnamon; and *Cinnamomum cassia* or *Cinnamomum Aromaticum*, also called Chinese Cinnamon, that are considered the main species. The latter is of lower value due to its less intense aroma and deeper color, while Ceylon Cinnamon is colored brown and has a gently scented aroma and a warm, sugary taste. Chinese Cinnamon differs from Ceylon Cinnamon also for the presence of a higher quantity of coumarin (0.8 to 10.63% for Chinese Cinnamon and approximately 2% for Ceylon Cinnamon), a substance that, if consumed in high doses, presents a risk of liver toxicity. *Cinnamomum*, beyond its use as an additive in cooking, is used to treat some diseases, such as diabetes mellitus, mainly due to the presence of cinnamaldehyde (65–80%) and eugenol (70–95%) derived from bark and leaves, respectively. Other substances with hypoglycemic activity are phenolic compounds, including quercetin, rutin, and catechins extracted from leaves and bark of *Cinnamomum* [54].

#### 3.4.1. Mechanisms of Action

**Figure 4:**
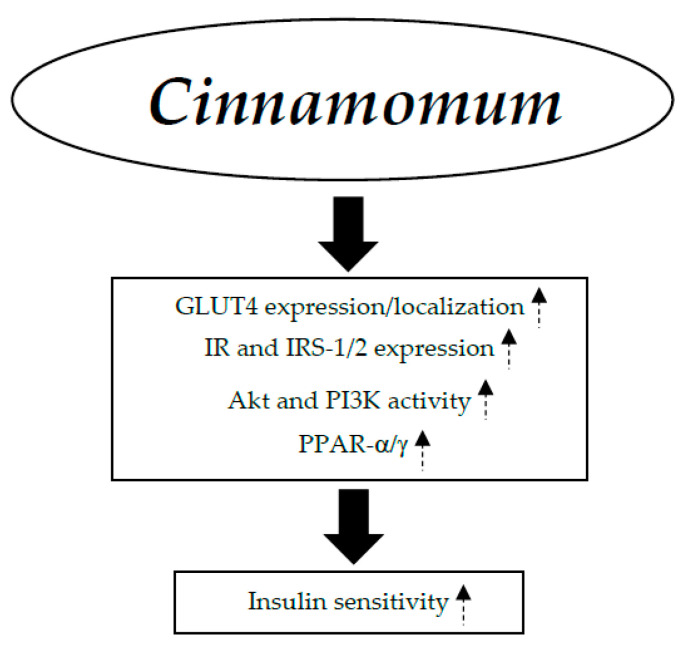
Glucose-lowering mechanisms of action of *Cinnamomum.* Akt: serine/threonine kinase; GLUT: glucose transporter; IR: insulin receptor; IRS-1/2: insulin receptor substrate type 1/2; PI3K: phosphatidylinositol 3-kinase; PPAR-α/γ: peroxisome proliferator-activated receptor-α/γ. 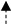: increase.

*Cinnamomum* has been shown to increase insulin sensitivity through a number of possible biochemical pathways, such as (1) stimulation of GLUT4 location and expression; (2) rise in expression of insulin receptor (IR), insulin receptor substrate type 1 (IRS-1), and insulin receptor substrate type 2 (IRS-2) [55,56]; (3) promotion of the activity of AKT and PI3K (phosphatidylinositol 3-kinase) [55,56]; and (4) enhanced activation of PPAR-α/γ [57] (Figure 4).

#### 3.4.2. Clinical Trials

Clinical trials that have shown how Cinnamomum varieties affect insulin sensitivity and glucose metabolism are summarized in Table 4.

A total of 60 T2DM patients received three different doses of *Cinnamomum cassia* (1, 3, or 6 g) or placebo daily for 40 days, after which there was a 20-day wash-out period. It was observed that FPG values were reduced by all doses of supplement in a similar manner (*p* < 0.05 for all doses) compared to initial values and by the lowest dose of nutraceutical (*p* < 0.05) following the wash-out phase in relation to values prior to the ingestion of *Cinnamomum cassia* [58].

Mang et al. enrolled 79 patients with T2DM in therapy with diet or hypoglycemic drugs who took *Cinnamomum cassia* powder 3 g/day or placebo. After 4 months of treatment, the supplement determined a significant reduction in FPG levels (*p* < 0.001) compared to initial values [59].

One study, comprising 25 postmenopausal T2DM patients treated with *Cinnamomum cassia* 1.5 g/day or placebo for 6 weeks, showed that FPG, FPI, HbA_1c_, HOMA-IR, composite index of insulin sensitivity (ISIcomp), and oral glucose insulin sensitivity (OGIS) were not modified after supplementation [60].

Another trial enrolled 15 women with polycystic ovary syndrome who consumed 1 g/day of cinnamon extract or placebo for 8 weeks. The supplement determined a significant decrease in FPG (*p* < 0.03) compared to pretreatment values. Cinnamon extract also ameliorated insulin sensitivity by increasing quantitative insulin sensitivity check index (QUICKI) (*p* < 0.03) and the Matsuda insulin resistance index (*p* < 0.05), as well as reducing HOMA-IR (*p* < 0.03), compared to baseline [61].

A sample of three lean healthy volunteers underwent 3 OGTT were supplemented with 5 g of *Cinnamomum cassia*, 5 g of placebo, or 5 g of the nutraceutical ingested 12 h before the trial. It was observed that AUC glucose was significantly decreased after OGTT with supplement (*p* < 0.05) and the one with *Cinnamomum cassia* ingested 12 h before the study (*p* < 0.05) compared to OGTT with placebo. In addition, the Matsuda insulin resistance index was elevated both following OGTT with supplement (*p* < 0.05) and the one with *Cinnamomum cassia* ingested 12 h before the trial (*p* < 0.05) compared to OGTT control [62].

In the study of Blevins et al., 60 T2DM patients received *Cinnamomum cassia* 1 g/day or placebo and did not exhibit any change in FPG, FPI, and HbA_1c_ levels after 3 months of supplementation [63].

A 3-month study comprising 72 adolescents with T1DM administered with cinnamon 1 g/day or placebo reported that HbA_1c_, total daily use of insulin or number of hypoglycemic episodes were not significantly different when comparing supplement and placebo [64].

In the study by Solomon and Blannin, eight healthy volunteers ingested 3 g/day of powdered spice from *Cinnamomum cassia* for 14 days or placebo for 20 days and were subjected to OGTT on days 0, 1, 14, 16, 18, and 20. The supplement significantly reduced PPG at 30 min on day 1 and 14 (*p* < 0.05) and glucose AUC at day 1 (−13.10%, *p* < 0.05) compared to control. It was also observed that Cinnamomum cassia, on day 14, decreased PPI at 30 min (*p* < 0.05) and insulin AUC (−27.10%, *p* < 0.05) compared to control, whereas it improved insulin sensitivity (*p* < 0.05) compared to day 0 and to all days in the control group [65].

On the other hand, it was demonstrated that, in 109 T2DM patients administered either their usual medications alone or together with 1 g/day of Cinnamomum cassia for three months, HbA_1c_ values were decreased in comparison to baseline (*p* < 0.001) and control (*p* < 0.04) [66].

In a group of 14 T2DM patients treated with cinnamon 1.5 g/day or placebo for 1 month, the supplement significantly reduced FPG levels (*p* < 0.05) compared to initial values [67].

Another trial reported that the administration of 2 g/day of *Cinnamomum cassia* powder or placebo for 3 months in 58 T2DM patients on oral hypoglycemic drugs significantly decreased FPG (*p* < 0.05) compared to initial values, and HbA_1c_ (*p* = 0.029) compared to placebo [68].

Markey et al. showed that the consumption of a high fat meal supplemented with 3 g *Cinnamomum zeylanicum* powdered or placebo in 9 healthy young subjects did not significantly change PPG levels after supplementation [69].

An 8-week study enrolled 44 patients with T2DM who were given *Cinnamomum zeylanicum* 3 g/day of or placebo. A significant reduction in FPG and HbA_1c_ (*p* < 0.05 for both) was found following the consumption of the supplement compared to baseline [70].

A randomized, placebo-controlled study evaluated the hypoglycemic effects of cinnamon at the dose of 3 g/day or 6 g/day for 3 months in 150 individuals with recently diagnosed T2DM. Both supplement dosages reduced in a similar way FPG (*p* < 0.001 for 3 and 6 g/day of cinnamon) and HbA_1c_ values *(p* < 0.005 for 3 g/day of cinnamon; *p* < 0.05 for 6 g/day of cinnamon) compared to those before taking cinnamon [71].

Another trial, performed on 66 Chinese patients with T2DM, confirmed the improvement of glucose metabolism markers after ingested different doses of cinnamon. Following 3 months of treatment with 120 mg/day or 360 mg/day of *Cinnamomum aromaticum* extract or placebo, both dosages lowered FPG (*p* < 0.01 for 120 and 360 mg/day of *Cinnamomum aromaticum* extract) and HbA_1c_ levels *(p* < 0.01 for 120 and 360 mg/day of *Cinnamomum aromaticum* extract) compared to initial values [72].

In contrast, two placebo-controlled studies demonstrated that consumption of *Cinnamomum cassia* 2 g/day for 2 months and ingestion of this supplement 12 g/day for 12 weeks did not alter FPG and HbA_1c_ in 70 patients with T2DM [73] as well as FPG, FPI and HbA_1c_ in 21 individuals with IGT [74].

However, a sample of 40 patients with T2DM not adequately controlled by oral hypoglycemic drugs took 1.5 g/day of a crude grind cinnamon for 3 months. The supplement significantly decreased FPG, random blood sugar (RBS), and HbA_1c_ (*p* < 0.01 for all) by comparison to the values before to the intervention [75].

Beejmohun et al. showed that the intake of Ceylon cinnamon hydro-alcoholic extract 1 g or placebo 30 min before the test meal in 18 healthy volunteers determined a reduction in PPG (*p* < 0.05) but not PPI following supplementation compared to placebo. In addition, glucose AUC 0–120 min was lowered by 14.80% without reach statistical significance (*p* = 0.15) whereas glucose AUC 0–60 min was significantly decrease (*p* < 0.05) after cinnamon consumption compared to placebo [76].

A randomized clinical trial enrolled 30 nondiabetic subjects who ingested 6 g/100 mL of cinnamon tea (*Cinnamomum burmannii*) immediately after OGTT or only the latter. It was observed that the supplement significantly decreased maximum PPG concentration (*p* = 0.040) and variation in maximum glucose concentration (*p* = 0.029), but not iAUC glucose (*p* = 0.084), compared to control [77].

The glucose-lowering activity of cinnamon was evaluated in two placebo-controlled studies including, respectively, 105 patients with type 2 diabetes treated with 1 g/day of this nutraceutical or blueberry for a period of 3 months [78] and 137 patients with FPG >110 mg/dL or 2h-PPG >140 mg/dL receiving 500 mg/day of aqueous extract of *Cinnamomum cassia*, dried by spraying for 2 months [79]. The supplement significantly reduced FPG (*p* = 0.006), 2h-PPG (*p* = 0.003), FPI (*p* = 0.019), HbA_1c_ (*p* = 0.010), and HOMA-IR (*p* = 0.007) in the first study [78] and lowered FPG (*p* < 0.005), 2h-PPG (*p* < 0.0001), FPI (*p* < 0.05), and HOMA-IR (*p* < 0.005) in the second trial [79], both compared to baseline.

Conversely, the administration of cinnamon 3 g/day or placebo in 44 T2DM patients did not change FPG, FPI, HbA_1c_, and HOMA-IR values after 8 weeks of supplementation [80].

Instead, in a sample of 116 individuals with metabolic syndrome given cinnamon 3 g/day or placebo 2.5 g/day for 16 weeks the supplement decreased FPG (*p* = 0.001), PPG (*p* = 0.030) and HbA_1c_ (*p* = 0.023) compared to initial values [81].

In a 12-week placebo-controlled trial, it was shown that the ingestion of cinnamon powder 1.5 g/day in 66 women with polycystic ovarian syndrome (PCOS) significantly decreased FPI (*p* = 0.024) and HOMA-IR (*p* = 0.014) compared to placebo [82].

One study included 140 T2DM patients that received, based on their baseline body mass index (BMI), 1 g/day of powdered cinnamon bark (BMI ≥ 27 and BMI < 27) or placebo (BMI ≥ 27 and BMI < 27) for a period of 3 months. It was observed that cinnamon significantly reduced FPG, HbA_1c_, FPI, HOMA-IR (*p* < 0.001 for all), and 2h-PPG (*p* = 0.008) when comparing to baseline, and patients with BMI ≥ 27 treated with the supplement showed greater change in glycemic markers [83].

In the trial of Kizilaslan and Erdem, 41 healthy subjects were given 1 g/day, 3 g/day, and 6 g/day of cinnamon, respectively, for 40 days. It was demonstrated that the highest dose of cinnamon significantly decreased FPG (*p* = 0.035) compared to before consumption of the supplement. A significant reduction in 2h-PPG (*p* = 0.028) at the dose of 1 g/day of cinnamon compared to the measurement on day 20 was also observed, as well as at the dose of 3 g/day (*p* = 0.018) and 6 g/day (*p* = 0.017), compared to baseline [84].

Another study, comprising 160 T2DM patients on oral hypoglycemic agents who took *Cinnamomum verum* 3 g/day or placebo for 3 months, showed that the supplement reduced FPG, HbA_1c_ (*p* = 0.001 for both), and HOMA-IR (−0.47, *p* = 0.006) compared to initial values [85].

A recent trial found that, in 36 T2DM patients who received OGTT immediately followed by 6 g/100 mL of aqueous cinnamon extract (*Cinnamomum burmannii*) ingestion or just OGTT, the supplement did not affect maximum glucose concentration (*p* = 0.527), glucose concentration variation (*p* = 0.873), or iAUC glucose (*p* = 0.834) when compared to the control group [86].

The hypoglycemic action of *Cinnamomum* in association with other nutraceuticals has also been investigated.

One study was conducted on 62 overweight or moderately obese individuals with prediabetes treated with a supplement composed of *Cinnamomum cassia*, chromium, and carnosine 1.2 g/day or placebo. After 4 months of treatment, the supplement significantly reduced FPG both to baseline (*p* = 0.026) and to placebo (*p* = 0.02) [87].

In contrast, 12 T2DM patients who were given 53.5 g/day of kanuka honey contained food-grade cinnamon 4.5 g, chromium polynicotinate 200 μg, and magnesium citrate 120 mg or unformulated honey showed no difference in FPG, FPI, or HbA_1c_ levels after 40 days of supplementation [88].

### 3.5. Gymnemic Acid [(Gymnema sylvestre (L.) Pers]

The leaves of *Gymnema sylvestre* (GS), a medicinal plant from the Asclepiadaceaea family, contain gymnemic acid, a compound of saponins, which has demonstrated hypoglycemic effects by delaying the absorption of glucose into the blood [89].

#### 3.5.1. Mechanisms of Action

**Figure 5:**
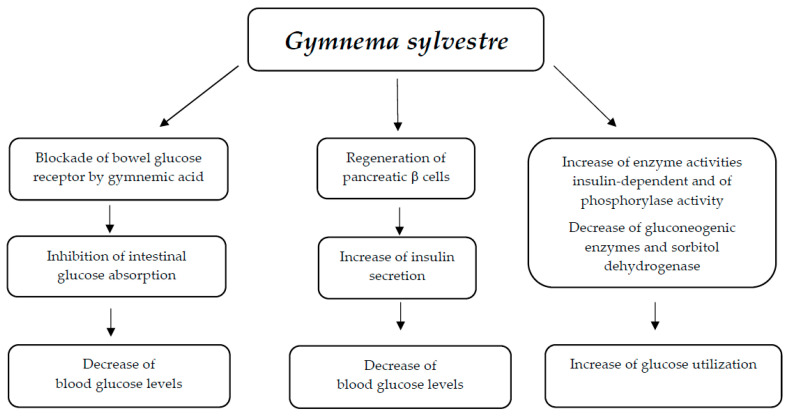
Hypoglycemic mechanisms of action of *Gymnema sylvestre*.

The receptor located on the taste buds is bound by gymnemic acid due to its similarity to glucose. As a result, the sugar present in the food does not activate the taste buds, leading to non-absorption of the sugar. The Na^+^-glucose symporters in the outer layer of the intestine are also targeted by gymnemic acid, which prevents glucose absorption [90]. Additionally, gymnemic acid induces hypoglycemic effects by enhancing insulin secretion and supporting the restoration of pancreatic β cells [91,92,93] (Figure 5). The hypoglycemic effects of gymnemic acid are also attributed to an increase in enzyme activities that are insulin-dependent, leading to a rise in glucose utilization. This includes increased activity of hexokinase, glycogen synthetase, glyceraldehyde 3-phosphate dehydrogenase, and glucose 6-phosphate dehydrogenase, as well as enhanced phosphorylase activity. Additionally, there is a reduction in gluconeogenic enzymes and sorbitol dehydrogenase [94]. Furthermore, gymnemic acid has a hypoglycemic effect as it controls the activity of incretin, stimulating the secretion and release of insulin [95].

#### 3.5.2. Clinical Trials

Various GS extracts demonstrated hypoglycemic action in multiple clinical trials (Table 5).

In one trial, six diabetic patients, who were not receiving any therapy, consumed, for fifteen days, 6 g/day of a preparation with ground GS leaves that had been shade-dried (10 g/100 mL). Both before and after the intervention, they underwent an OGTT. According to Khare et al. [96], the supplement significantly decreased FPG (*p* < 0.02,) and PPG at 30 min (*p* < 0.05), and 2 h (*p* < 0.01) following OGTT in comparison to the control.

Later research revealed that taking 400 mg/day of ethanol extract of GS leaves (GS4) in capsule form for 18–20 months reduced FPG and HbA_1c_ (*p* < 0.001 for both) in comparison to baseline and increased insulin levels during fasting and post-prandial (*p* < 0.01 for both) in comparison to diabetics not taking a supplement [97].

Twenty-seven type 1 diabetic patients who were insulin-dependent were also administered the supplement utilized in the Baskaran et al. [96] trial for a duration of six to thirty months. At the conclusion of the treatment, a not statistically significant reduction in FPG readings and a decrease in the daily requirement of insulin of approximately 50% were seen, compared to initial values. This reduction is likely due to the supplement’s ability to support the renewal of the remaining pancreatic β cells. Furthermore, research has demonstrated that throughout the first 6 to 8 months of treatment, GS leaf extract significantly reduced HbA_1c_ levels (*p* < 0.001) in comparison to baseline, and this reduction persisted until the completion of the regimen. HbA_1c_, however, was greater than those of the control group. Furthermore, compared to diabetics not taking supplements, the supplementation resulted in a considerable rise in fasting C-peptide concentrations (+0.08 mg/dL, +76.20%, *p* < 0.001), which suggests that pancreatic β cell activity has improved [98].

After receiving a new GS leaf extract with a high molecular weight [OSA (Om Santal Adivasi)] 1 g/day for 60 days, a group of 11 T2DM patients showed a significant reduction in their baseline levels of FPG (*p* < 0.005) and PPG (*p* < 0.02). Additionally, compared to baseline, there was a documented increase in C-peptide (*p* < 0.05) and insulin (*p* < 0.001) [99].

Furthermore, in isolated human islets of Langerhans, it was shown that OSA increases insulin secretion [100,101].

A different study found that giving 250 mg of GS leaf extract in capsule form to 58 T2DM patients for three months significantly reduced their levels of FPG (*p* < 0.005) and PPG (*p* < 0.001), and concurrently reduced their HbA_1c_ value (*p* < 0.001) in comparison to baseline. It was also observed that, after GS supplementation, along with a decrease in HOMA-IR (*p* = 0.36) and a rise in the homeostatic model assessment of β cell function (HOMA-B) (*p* = 0.26), the glucose/insulin ratio had statistically significant decreased (*p* = 0.01) compared to initial values [102].

In a sample of 32 T2DM patients, Li et al. found that giving 1 g/day of GS leaf powder in rigid gelatin capsules for 30 days resulted in a substantial reduction in FPG (*p* < 0.05) compared to values before the intervention [103].

The effects of giving GS leaf extract to IGT patients not using hypoglycemic medications have just been investigated. Thirty subjects were recruited and split into two groups of fifteen each, each of which was given 300 mg of GS powdered leaves as capsule twice a day for 12 weeks, or a placebo. Additionally, these individuals had an OGTT both before and after taking the supplements. According to the study’s findings, the GS leaf extract consumption led to a significant drop in HbA_1c_ (*p* = 0.003) and PPG at two hours after OGTT (*p* = 0.025) compared to baseline, which increased insulin sensitivity [104].

**Table 5:** Summary of clinical trials on glucose-lowering activity of *Gymnema sylvestre*.

First Author and Year	Participants	Therapy and Duration	Findings
Khare, A.K. (1983) [96]	Diabetic patients (n = 6)	6 g/day decoction of powdered GS leaves shade-dried 15 days	FPG decrease, PPG at 30 min and 2 h after OGTT decrease
Baskaran, K. (1990) [97]	T2DM patients (n = 22)	400 mg/day GS_4_ 18–20 months	FPG and HbA_1c_ decrease Insulin increase in fasting and post-prandial state
Shanmugasundaram, E.R. (1990) [98]	Type 1 diabetic patients (n = 27)	400 mg/day GS_4_ 6–30 months	FPG and HbA_1c_ decrease Less insulin requirement Fasting C-peptide increase
Al-Romaiyan, A. (2010) [99]	T2DM patients (n = 11)	1 g/day OSA 60 days	FPG and PPG decrease insulin and C-peptide increase
Kumar, S.N. (2010) [102]	T2DM patients (n = 58)	250 mg/day capsule of GS leaf extract 3 months	FPG, PPG and HbA_1c_ decrease Insulin resistance decrease
Li, Y. (2015) [103]	T2DM patients (n = 32)	1 g/day capsule of GS leaf extract 30 days	FPG decrease
Gaytán Martínez, L.A. (2021) [104]	IGT patients (n = 30)	600 mg/day capsule of GS leaf extract 12 weeks	HbA_1c_ decrease PPG 2 h after OGTT decrease

FPG, fasting plasma glucose; GS, *Gymnema sylvestre*; GS_4_, ethanol extract of GS leaves; HbA_1c_, glycated hemoglobin; IGT, impaired glucose tolerance; OGTT, oral glucose tolerance test; OSA, Om Santal Adivasi; PPG, post-prandial plasma glucose; T2DM, type 2 diabetes mellitus.

### 3.6. Ilex paraguariensis (L.) Pers

Yerba mate, or *Ilex paraguariensis* (St. Hill, Aquifoliaceae), is a tree everlasting native to Paraguay, Argentina and southern Brazil. The plant’s dried and crushed leaves are utilized to make the local beverages known as maté, tererê, and chimarrão, which are distinguished by their bitter flavor and stimulating qualities. Brazil’s mate tea is another infusion that is drank in the form of dried, powdered, and roasted *Ilex paraguariensis* leaves. It can be consumed hot or cold and is prized for its delicate and pleasant aroma [105].

Because it contains a variety of bioactive substances, such as polyphenols (chlorogenic and gallic acids, catechins), methylxantines (caffeine and theobromine), flavonoids, saponins, amino acids, minerals, and vitamins, *Ilex paraguariensis* has demonstrated potential health benefits for humans, including lowering of body weight, reducing effect on glucose and cholesterol, and strong antioxidant activity [106].

#### 3.6.1. Mechanisms of Action

**Figure 6:**
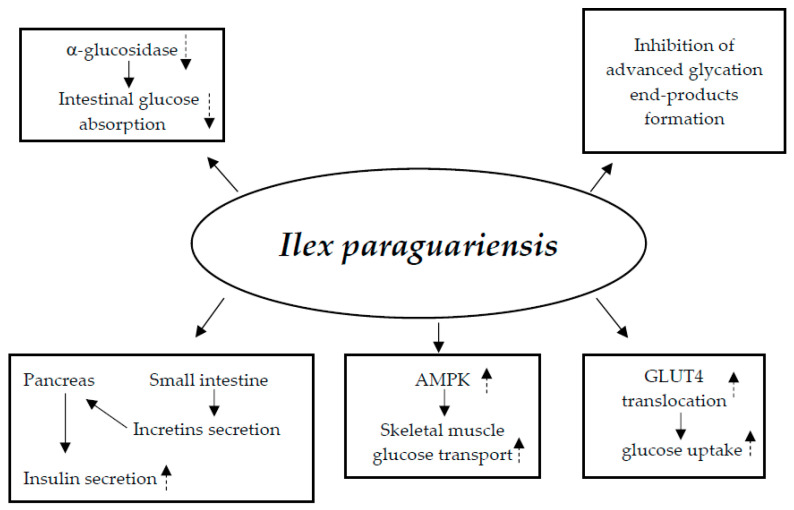
Glucose-lowering mechanisms of action of *Ilex paraguariensis*. AMPK, adenosine monophosphate-activated protein kinase; GLUT, glucose transporter; 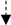, decrease; 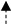, increase.

Studies on animals have demonstrated the potential hypoglycemic processes of bioactive ingredients found in Ilex paraguariensis, particularly polyphenols. These substances have a decreasing effect on glucose: (1) stimulating the release of insulin in the pancreas and inducing the secretion of incretins in the small bowel; (2) activating the transport of glucose in skeletal muscle through AMPK activation; (3) stimulating the translocation of GLUT4 and the absorption of glucose; (4) suppressing α-glucosidase, thereby decreasing the entry of glucose in the gut; and (5) repressing the formation of advanced glycation end-products [107,108,109] (Figure 6).

#### 3.6.2. Clinical Trials

There has been conflicting information regarding *Ilex paraguariensis*’s hypoglycemic effects from human trials (Table 6).

According to one study, drinking 200 mL of maté tea (12.5 mg/mL) daily for two months did not change the FPG levels in either group in 42 normolipidemics and 18 hyperlipidemics [110].

On the other hand, in the study by Klein et al., 29 patients with type 2 diabetes and 29 patients with prediabetes drank roasted maté tea for 2 months, either alone or in conjunction with dietary counseling or dietetic counseling. A total of 330 mL of boiling water was combined with 6.6 g of yerba mate leaves to prepare maté tea, which was consumed three times a day for breakfast, lunch, and dinner. The authors showed that the supplement significantly reduced FPG after 2 months (*p* < 0.05) and HbA_1c_ after 20 days and 2 months (*p* < 0.05 for both) in comparison with baseline values, only in patients with T2DM [111].

Alternatively, Kim et al. assessed the impact of a dried green *Ilex paraguariensis* leaves powdered extract on FPG in 46 overweight participants who were given a placebo or 334 mg/day of supplement for a period of 6 weeks. Both groups’ FPG levels showed no discernible changes at the conclusion of the treatment [112].

Still, the findings of a different study partially corroborated those of Klein et al. [110]. For 60 days, 330 milliliters of roasted maté tea was consumed by a group of 11 T2DM and 11 prediabetic individuals thrice daily, at breakfast, lunch, and supper. The preparation of the maté tea followed Klein et al.’s research instructions. The supplement significantly lowered HbA_1c_ and FPG (*p* < 0.05 for both) in T2DM patients after 60 days, and it also reduced HbA_1c_ (*p* < 0.05) in prediabetics after 40 days, in comparison to initial values [113].

Conversely, in 33 obese women who ingested maté extract 3g/day or placebo for 6 weeks, the supplement did not alter FPG [114].

Several studies investigated Ilex paraguariensis’s hypoglycemic effect in combination with other supplements.

Derosa et al. assessed the effects on glucose parameters of a nutraceutical compound containing 500 mg of Ilex paraguariensis, 50 mg of white mulberry, 1 mg of I-deoxinojirimcina, and 100 μg of chromium picolinate in 137 patients, IFG or IGT, who were randomly assigned to consume 1 tablet of supplement or placebo at breakfast for three months. The nutraceutical product significantly lowered FPG and HOMA index in comparison to the initial values (*p* < 0.05 for both) and the placebo (*p* < 0.05 for both). Furthermore, following supplementation, M value (defined as the average glucose infusion rate, over a period of 80–120 min from the start of the insulin infusion during euglycemic hyperinsulinemic clamp), was higher than baseline and placebo (*p* < 0.05 for both). Additionally, nutraceutical compound was assumed to be beneficial, and it was seen that a group of patients equal to 67% regained normal insulin sensitivity, and the remaining ones had a rise in M value against placebo [115].

The effectiveness of a nutraceutical containing 1000 mg of *Ilex paraguariensis*, 50 mg of white mulberry, 1 mg of I-deoxinojirimcina, and 100 μg of chromium picolinate on glycemic status was examined by the same authors in 143 IFG or IGT patients who were given one tablet of a nutraceutical or a placebo at breakfast for three months. Following supplementation, there was a significant drop in FPG, PPG, HbA_1c_, and HOMA index when compared to initial values (*p* < 0.05 for all) and placebo (*p* < 0.05 for all). In addition, M value increased after nutraceutical administration (*p* < 0.05) in comparison to baseline. It was also observed that 22% of patients had an improvement in their M value with the supplement compared to a placebo, while the remaining came back with a M value within the range of normal insulin sensitivity [116].

### 3.7. Momordica charantia (L.)

*Momordica charantia*, a climber plant in the Cucurbitaceae family, is grown in China, India, East Africa, Central America, and South America. It is also referred to as bitter melon or bitter gourd [117,118]. Charantin; polypeptide-P; vicine; momordin; and momordin’s derivatives, comprising momordenol, momordicilin, momorcharin, and momordicin, are among the phytochemical constituents that have been isolated from bitter melon fruits, seeds, leaves, stems, pericarps, endosperm, callus tissue, and cotyledons and have been shown to have hypoglycemic potential [119,120,121].

#### 3.7.1. Mechanisms of Action

**Figure 7:**
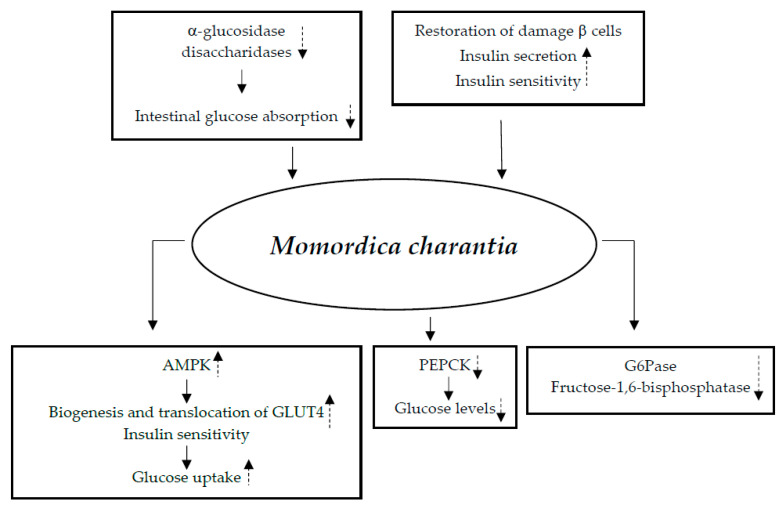
Glucose-lowering mechanisms of action of *Momordica charantia*. AMPK: adenosine monophosphate-activated protein kinase; GLUT: glucose transporter; G6Pase: glucose-6-phosphatase; PEPCK: phosphoenolpyruvate carboxykinase. 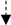: decrease; 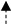: increase.

Numerous modes of action for the hypoglycemic effects of Momordica charantia have been hypothesized based on extensive research conducted in animal models. According to reports, the plant’s extracts (1) improve the activity of AMPK, which upregulates the biogenesis and translocation of GLUT4 and, therefore, glucose absorption, apart from enhancing insulin sensitivity [122]; (2) lower glucose levels by inhibiting the expression of PEPCK [123]; (3) inhibit the G6Pase and fructose 1,6-bisphosphatase enzymes [124,125]; (4) inhibit the activities of α-glucosidase and disaccharidases, thus lowering gut glucose absorption [122,126]; and (5) participate in the recuperation of injured β cells, promote insulin production, and raise peripheral insulin sensitivity [122] (Figure 7).

#### 3.7.2. Clinical Trials

*Momordica charantia*’s hypoglycemic effects were demonstrated in many clinical trials (Table 7).

Nine individuals with type 2 diabetes, six of whom had juvenile diabetes, one with adulthood-onset, and two without symptoms, received a dose of vegetable insulin (v-insulin) subcutaneously derived from *Momordica charantia* fruit that is similar to animal insulin or placebo. Patients with mild diabetes and FPG greater than 180 mg/dL, those with moderate diabetes and FPG less than 180 and greater than 250 mg/dL, and subjects with severe diabetes and FPG equal to or greater than 250 mg/dL were given 10, 20, and 30 units, respectively. A decrease in FPG was found, ranging from 21.50% to 24.80%, 30–60 min after v-insulin administration, and a reduction by 49.20% was noted 4 h following its injection [127].

In one trial, nine diabetic patients who were not on insulin therapy were subjected to three OGTT tests: the first was a conventional test, the second was a test using 50 mL of bitter melon fruit extract soluble in water, and the last one was performed following daily consumption of 0.23 kg of fried bitter melon fruit for a period between 8 and 11 weeks.

Bitter melon fruit extract soluble in water significantly decreased PPG after 30 min (*p* < 0.05), 60 min, and 90 min (*p* < 0.001 for both); and the mean glucose iAUC after 0–30 min (*p* < 0.05), 30–60 min (*p* < 0.01), and 60–90 min (*p* < 0.001). The daily consumption of fried bitter melon fruit also reduced the mean glucose iAUC after 0–30 min (*p* < 0.01), 30–60 min, and 60–90 min (*p* < 0.05 for both) compared to conventional test. But compared to fried bitter melon fruit, bitter melon fruit extract soluble in water was better at enhancing glucose tolerance [128].

A saline solution of polypeptide-P derived from fruits, seeds, and tissue culture of *Momordica charantia* was given via subcutaneous route to 19 diabetic patients, including 11 subjects with diabetes onset in youth and 8 in adulthood, at the dose of 10 units for FPG under 180 mg/dL, 20 units for FPG between 180 and 250 mg/dL, and 30 units for FPG equal to or greater 250 mg/dL. When administered to juvenile diabetes patients, polypeptide-P dramatically lowered FPG at specific time intervals in relation to both the FPG values of the treated subjects and the controls, whereas in patients with diabetes onset in adulthood polypeptide-P decreased FPG after 1 h and 6 h (*p* < 0.05 for both) compared to control [129].

For a period of seven days, eight patients with adulthood-onset diabetes were given 50 mg/kg of entirely dried and pulverized *Momordica charantia* fruit after breakfast and dinner, together with milk. This resulted in a significant decrease in PPG levels at certain time intervals compared to initial values [130].

One study enrolled 18 adult patients with diabetes who drank 100 mL of *Momordica charantia* juice or distilled water half an hour prior to OGTT, and 13 of them had a noteworthy enhancement in their glucose tolerance [131].

A sample of 50 subjects with mild-to-moderate type 2 diabetes took 2 g of *Momordica charantia*, dried whole fruit powder, or placebo and showed no change in FPG and PPG following 2 and 4 weeks of supplementation. Reevaluating the plant’s hypoglycemic potential was recommended using the uncooked fruit or an alternative way of extracting its contents [132].

Another trial was conducted on 15 patients with T2DM subjects who were non-insulin-dependent; they were treated with metformin 1 g/day, glibenclamide 10 mg/day, or a mixture of the two drugs for one week, and, afterward, they were treated with half their dosage of these hypoglycemic agents, together with 400 mg/day of fruit extract from *Momordica charantia*, for one week. It was observed that the supplement combined with the drugs at half dosage reduced FPG (*p* > 0.01 for supplement plus metformin; *p* > 0.001 for supplement plus glibenclamide; *p* > 0.001 for supplement plus metformin and glibenclamide) and PPG (*p* < 0.001 for supplement plus metformin; *p* > 0.001 for supplement plus glibenclamide; *p* > 0.001 for supplement plus metformin and glibenclamide) to a greater extent than drugs at full dosage without supplement [133].

A group of forty patients with inadequately controlled and recently diagnosed type 2 diabetes were given 3 g/day of Momordica charantia or placebo, and after three months, it was found that FPG and HbA_1c_ did not change following the supplementation. [134].

In a six-month study including 50 subjects with T2DM non-insulin-dependent who received either 55 mL of *Momordica charantia* juice or 4 mg of rosiglitazone daily, the supplement did not affect glycemia compared to controls [135].

Five overweight patients took either 50 mg/kg body weight or 100 mg/kg body weight of freeze-dried *Momordica charantia* juice before OGTT. There were no differences in insulin and plasma glucose in the fasting and post-prandial phases [136].

Three dosages of dried *Momordica charantia* leaves (60, 80, and 100 mg/kg/day) or a placebo were administered to 40 patients recently diagnosed with type 2 diabetes, and a usual morning meal was then served. Supplementation at the 100 mg/kg/day dose increased insulin secretion (*p* = 0.012) and lowered mean plasma glucose at 15 min (*p* = 0.04) and mean PPG over the 0 to 4 h interval after administration more than the other two doses and placebo [137].

The consumption of three dosages of *Momordica charantia* (500 mg/day, 1 g/day, and 2 g/day, respectively) or metformin 1 g/day for four weeks in 120 subjects with a recent diagnosis of type 2 diabetes did not reduce FPG after supplementation. After OGTT, there were no discernible variations in 2h-PPG levels with the three doses of Momordica charantia [138].

For three months, 42 participants with metabolic syndrome received 4.8 g of freeze-dried wild bitter melon powder per day. During the supplementation, and for three months after its conclusion, the patients were observed monthly. Variations in insulin resistance markers (logHOMA, QUICKI, and McAuley values) were found, resulting in increased insulin sensitivity [139].

One trial was performed on 95 patients with type 2 diabetes who were given 2 and 4 g of bitter melon or glibenclamide 5 mg daily for a period of 10 weeks. Fasting plasma glucose and HbA_1c_ were lowered by glibenclamide (*p* < 0.003 for FPG; *p* < 0.005 HbA_1c_) more than bitter melon (FPG: *p* ≤ 0.05 for 2 g/day of nutraceutical; *p* < 0.04 for 4 g/day of nutraceutical; HbA_1c_: *p* ≤ 0.05 for 2 g/day of nutraceutical; *p* ≤ 0.02 for 4 g/day of nutraceutical), whereas 2h-PPG (*p* < 0.03) was reduced only by glibenclamide compared to initial values [140].

A two-month study enrolled 52 subjects with prediabetes who ingested 2.5 g/day of dry bitter gourd or placebo. The supplement significantly lowered FPG, and this reduction was largest among patients who had higher FPG values at baseline [141].

*Momordica charantia* fruit powder (2 g/day) or a placebo was taken by 25 patients with type 2 diabetes for three months. It was found that HbA_1c_, AUC glucose (*p* < 0.05 for both), and 2h-PPG (*p* < 0.01) were reduced, whereas AUC insulin, insulinogenic index, and Stumvoll index were raised (*p* < 0.05 for all) after supplementation compared to starting values [142].

A sample of 90 patients with T2DM receiving 2.38 g per day of bitter melon extract or placebo for 3 months exhibited a significant reduction in FPG (*p* = 0.014) and HOMA-IR (*p* = 0.017) following supplementation, compared to initial values [143].

*Momordica charantia* combined with other supplements was also studied.

In one trial, which included 60 patients with type 2 diabetes who were non-insulin-dependent, a mixture of powdered *Momordica charantia* fruit, fenugreek seeds, and jamun seeds, uncooked or cooked, was consumed at a dose of 1g/day for 1.5 month and then 2 g/day for 1.5 month. Both dosages reduced FPG and PPG, whether in the uncooked (*p* < 0.05 for both) or cooked form of the mixture (1 g/day: *p* < 0.05 for FPG; *p* < 0.01 for PPG; 2 g/day: *p* < 0.05 for both), compared to control, and the higher dose resulted in a greater reduction in FPG and PPG levels. Additionally, the proportion of participants receiving oral hypoglycemic agents dropped from 82% prior to supplementation to 67% with 1 g/day of nutraceuticals combination, and 33% with 2 g/day of the supplement [144].

### 3.8. Morus

The family Moraceae includes the genus Morus, commonly referred to as mulberries. According to Kumar and Chauhan [145], *Morus alba* (L.) Pers (white mulberry), *Morus nigra* (L.) Pers (blackberry), and *Morus rubra* (L.) Pers (red berry) are the most well-known species and are especially widespread in East, South and Southwest Asia, North Africa, and South Europe.

Because of the antioxidant qualities of these substances, the species *Morus*, which has a high concentration of phenolic compounds, including flavonoids and anthocyanins, is used to prevent hepatic and renal diseases, joint injury, and aging [146].

The presence of sugar-mimicking alkaloids, such as 1,4-dideoxy-1,4-imino-D-arabinitol, 1-deoxynojirimycin (DNJ), and 1,4-dideoxy-1,4-imino-D-ribitol, is widely distributed in the species Morus, which makes it effective in treating type 2 diabetes [147,148]. The mulberry tree’s roots, leaves, and fruits are often the portions that are investigated for potential medicinal uses [149].

#### 3.8.1. Mechanisms of Action

**Figure 8:**
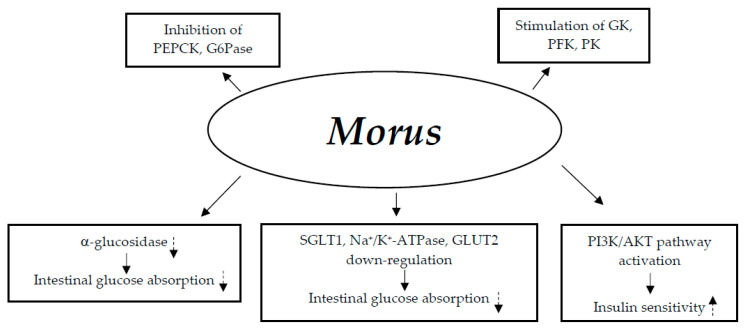
Hypoglycemic mechanisms of action of *Morus*. G6Pase: glucose-6-phosphatase; GLUT: glucose transporter; GK: glucokinase, PEPCK: phosphoenolpyruvate carboxykinase; PFK: phosphofructokinase, PK: pyruvate kinase; SGLT1: sodium glucose co-transporter 1; PI3K/AKT: phosphatidylinositol 3-kinase/serine/threonine kinase. 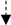: decrease; 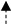: increase.

The hypoglycemic activity of Morus species may be caused by the following possible mechanisms: (1) decreased glucose absorption in the gut through suppression of α-glucosidase activity and downregulation of bowel sodium glucose co-transporter 1 (SGLT1) and Na^+^/K^+^-ATPase, as well as GLUT2 mRNA and protein expression; (2) increased insulin sensitivity through stimulation of the insulin signaling PI3K/AKT pathway; and (3) inhibition of gluconeogenic enzymes (PEPCK and G6Pase) and activation of glycolytic enzyme activities (glucokinase (GK), phosphofructokinase (PFK), and pyruvate kinase (PK)) [150,151] (Figure 8).

#### 3.8.2. Clinical Trials

Controversial results from clinical trials have been documented in the literature regarding the impact of Morus on glucose metabolism (Table 8).

Twenty-four healthy participants in the Kimura et al. trial were given powdered leaves of the mulberry (*Morus alba*) plant that had been supplemented with 0.4, 0.8, or 1.2 g of DNJ. An oral sucrose load was subsequently administered. When compared to a placebo, the nutraceutical containing DNJ at the dose of 0.8 or 1.2 g was found to lower PPG and PPI levels (*p* < 0.05 for both) 60 min after the sucrose load. In addition, the intake of 3.6 g/day of DNJ-enriched mulberry powder leaves or placebo did not alter the FPG levels in 24 healthy people after 38 days [150].

In another study, 10 T2DM patients and 10 controls consumed a supplement with 1 g plus sucrose 75 g or placebo for one week in order to assess the impact of extract from mulberry (*Morus alba*) leaves on PPG. During the course of the first 120 min of the trial, the supplement significantly reduced PPG in comparison to the placebo in both the controls (*p* = 0.005) and the diabetics (*p* = 0.002). Furthermore, at the conclusion of the study, the blood glucose reduction was lower with nutraceuticals compared to placebo [152].

After a carbohydrate tolerance test (200 g of boiling white rice), Asai et al. examined the effects on PPG and PPI of only one ingestion of leaf extract from mulberry (*Morus alba*) enhanced with DNJ at a dose of 3, 6, or 9 mg or placebo in 12 dysglycemia subjects. PPG levels were generally lowered by the supplement in a dose-dependent way. The groups that received leaf extract from mulberry supplemented with 6 and 9 mg DNJ showed a substantial drop in PPG levels (*p* < 0.05 m for both) 30 min after the meal test compared to placebo.

After 30 min, the subjects who received 3, 6, and 9 mg DNJ of the supplement had considerably lower post-prandial insulin levels (*p* < 0.05 for all) than the placebo group. Additionally, it was noted that, after 12 weeks of supplementation, 76 dysglycemic individuals who consumed 18 g/day of leaf extract from mulberry boosted with DNJ or a placebo did not have any changes in FPG, FPI, HbA_1c_, or glycated albumin (GA). Nonetheless, the supplement elevated serum 1,5-anhydroglucitol (1,5AG), a marker of post-prandial glucose levels management, at 4, 8, and 12 weeks (*p* < 0.05 for all) in comparison to starting values, as well as at 8 and 12 weeks (*p* < 0.05 for both) in comparison to placebo [153].

The effectiveness of a gelatine including 3.3 g of leaf extract from *Morus alba* (MLE) on PPG and PPI was investigated in one trial involving 10 patients with type 2 diabetes in therapy using sulfonylureas or not and 10 healthy participants. Thirty minutes after supplement administration, patients with T2DM showed a substantial drop in PPG and PPI (*p* < 0.05 for both) as compared to placebo. These parameters showed a similar trend (*p* < 0.05 for both) in T2DM patients who were not being treated or were receiving sulfonylurea treatment. Furthermore, thirty minutes after consuming MLE compared to placebo, a substantial decrease in PPG and PPI (*p* < 0.05 for both) was found in healthy volunteers [154].

In the Chung et al. trial, fifty healthy participants were given a drink that contained 75 g of dissolved powdered maltose in water, along with 0 (placebo), 1.25, 2.5, or 5 g of aqueous extract from mulberry (*Morus alba*) leaves (MLAE). Following the maltose load, it was demonstrated that PPG was reduced after ingesting 2.5 g of MLAE at 30 min (*p* = 0.0137) and 5 g of MLAE at 60 min (*p* = 0.0423) [155].

In 38 patients with IFG receiving 5 g of supplementation daily, researchers evaluated the impact of MLAE on PPG, PPI, and post-prandial C-peptide. These markers were found to be significantly reduced following carbohydrate loading, compared to placebo, at 30 min (*p* = 0.0003 for PPG, *p* = 0.0005 for PPI, and *p* = 0.0096 for post-prandial C-peptide) and 60 min (*p* = 0.0325 for PPG, *p* = 0.035 for PPI, and *p* = 0.0156 for post-prandial C-peptide). This was observed after 4 weeks of treatment. Additionally, after supplementation compared to placebo, there was a decrease in the area under the curve (AUC) for post-prandial C-peptide (*p* = 0.059) and PPI (*p* = 0.0207) [156].

An investigation conducted in 2015 found that giving 70 mL of either regular black tea or mulberry (*Morus alba*) leaf tea to 48 T2DM patients, along with a standardized breakfast, resulted in a decrease in FPG (*p* = 0.055) and PPG (*p* < 0.001) 90 min after the drinking of the mulberry tea compared to the regular one [157].

The study conducted by Sukriket et al. revealed that, in a group of 14 non-diabetic participants, the supplement, when taken 30 min before OGTT, significantly reduced PPG at 30 min (*p* = 0.04) compared to controls. The subjects drank water or mulberry (*Morus alba*) leaf tea powder (2 g) [158].

Additionally, the impact of MLE on HbA_1c_ and self-checking of blood sugar (SMBG) was assessed in seventeen patients with type 2 diabetes receiving a placebo or 3 g/day of supplement for three months. Postprandial SMBG was significantly lower in the MLE group at the end of treatment compared to baseline and to placebo (*p* < 0.05 for both). HbA_1c_ decreased after supplementation relative to baseline (*p* = 0.079), without reaching statistical significance [159].

In a further study, eighty-five healthy subjects were randomized to receive one dosage of 50 g of sucrose blended with mulberry powdered leaves at 0, 6, 12, and 18 mg of DNJ. Thirty minutes after the carbohydrate load, 6 mg of DNJ was shown to dramatically reduce PPG (*p* < 0.001) compared to control. 1-Deoxynojirimycin at 12 and 18 mg reduced PPG in comparison to the control at 30 (*p* < 0.001 for DNJ at 12 mg; *p* < 0.001 for DNJ at 18 mg) and 60 (*p* < 0.05 for DNJ at 12 mg; *p* < 0.001 for DNJ at 18 mg), as well as in comparison to DNJ at 6 mg at 30 and 60 min. These results demonstrated that the DNJ dose of 18 mg lowered PPG levels more than other doses. Nevertheless, DNJ at 12 mg proved to be the most favorable dose in regard to reducing PPG levels since it had a lower incidence of gastrointestinal adverse effects than DNJ at 18 mg. These investigators also enlisted 54 patients with obesity who received nutritional counseling or 36 mg/day of mulberry DNJ prior to primary meals. The patients had FPG values between 100 and 140 mg/dL and/or 2h-PPG between 140 and 199 mg/dL. When compared to baseline results, the supplement significantly reduced FPG and HbA_1c_ (*p* < 0.05 for both) after 12 weeks of treatment [160].

The effectiveness of standardized MLAE with 5% DNJ on post-prandial glucose and post-prandial insulin was investigated by Thondre et al. in 38 healthy subjects who were given either 250 mg of the supplement or 75 g of sucrose alone as a placebo. Following supplementation, compared to placebo, there was a substantial decrease in PPG at 15 (*p* < 0.001), 30 (*p* < 0.001), 45 (*p* = 0.008), and 120 min (*p* < 0.001), as well as in PPI at 15 (*p* < 0.001), 30 (*p* < 0.001), 45 (*p* < 0.001), 60 (*p* = 0.001), and 120 min (*p* < 0.001). Additionally, the supplement significantly decreased the maximum blood sugar and insulin levels (*p* < 0.001 for both) in comparison to placebo, as well as the average glucose and insulin iAUC at 60 (*p* < 0.001 for both), 90 (*p* < 0.001 for both), and 120 min (*p* = 0.001 for glucose iAUC; *p* < 0.001 for insulin iAUC) [161].

Momeni et al. included 100 T2DM patients who received a three-month treatment of 9 mL/day of a 4% hydro-alcoholic extract from *Morus nigra* leaves or placebo in water. According to observations, the supplement reduced HbA_1c_ and FPG (*p* < 0.001 for both) in comparison to baseline levels [162].

Studies on the impact of Morus in association with other supplements have also been performed.

According to prior reports, one study demonstrated the hypoglycemic action of a mixture containing extract from mulberry leaves, extract from banaba leaves, and powdered Korean red ginseng on several indicators [26].

In a different trial, 40 individuals with dyslipidemia consuming a supplement for 12 weeks, or a placebo, were assessed for the impact on glucose parameters of a combination including 43 mg of *Morus alba*, 129 mg of *Crataegus pinnatifida*, 86 mg of *Alisma orientalis*, 86 mg of *Stigma maydis*, 43 mg of *Ganoderma lucidum*, and 43 mg of *Polygonum multiflorum*. After supplementation, there was a substantial drop in HbA_1c_ at the conclusion of treatment (*p* < 0.01) compared to starting values [163].

In an eight-week study, two distinct nutraceutical associations (A and B) were administered to twenty-three hypercholesterolemic individuals who were not taking statins. In combination A, there was 10 mg of policosanol, 200 mg of red yeast rice, 500 mg of berberine, 0.5 mg of astaxanthin, 200 μg folic acid, and 2 mg of coenzyme Q10, while in combination B, there was 531.25 mg of berberine, 220 mg of powdered red yeast rice, and 200 mg of *Morus alba* leaf extract. Combination B was found to significantly lower FPG, FPI, HbA_1c_, and HOMA index compared to initial values (*p* = 0.0001 for FPG; *p* = 0.006 for FPI; *p* = 0.006 for HbA_1c_ and HOMA index) and to combination A (*p* = 0.0001 for FPG; *p* = 0.02 for FPI; *p* = 0.03 for HbA_1c_; *p* = 0.002 for HOMA index) [164].

As previously mentioned, the impact of the combination of white mulberry, Ilex paraguariensis, and chromium picolinate on glycemic control has also been investigated [115,116].

### 3.9. Olea europaea (L.)

Likely native to Asia Minor and Syria, the olive plant (*Olea europaea*) is a member of the Oleaceae family. Numerous biologically active constituents, including polyphenols, oleuropein, hydroxytyrosol, tyrosol, ligstroside, lignans, and flavonoids, are found in the leaves and olives, the fruits, of this plant, and they are are often employed in conventional herbal therapy, especially in the Mediterranean area. These substances may have anti-inflammatory, antihypertensive, anti-atherogenic, antioxidant, hypoglycemic, and hypocholesterolemic effects [165,166]. Oleuropein, a naturally occurring compound belonging to secoiridoid group, is the primary active ingredient. It has been linked to the enhancement of glucose homeostasis both in vitro and experimental model studies [167,168]. In addition, oleuropein is a significant component of olive leaves, and it is the primary ingredient in olive leaf extract (OLE), a supplement that is available [169].

#### 3.9.1. Mechanisms of Action

**Figure 9:**
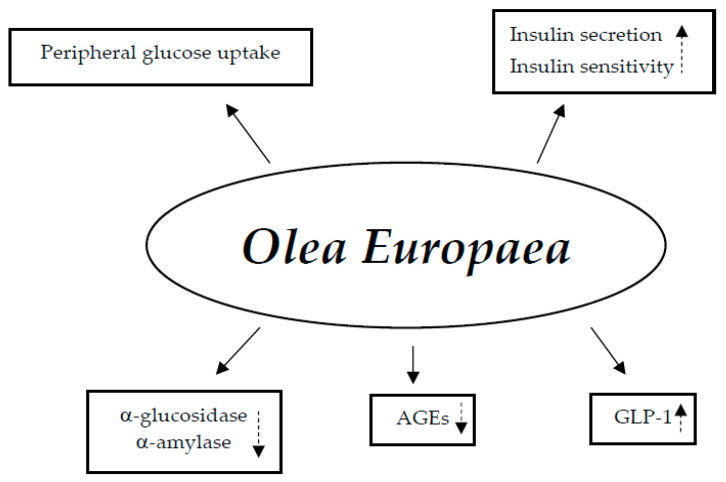
Potential hypoglycemic mechanisms of action of *Olea europaea*. AGEs; advanced glycation end-products; GLP-1: glucagon-like peptide-1. 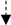: decrease; 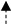: increase.

Studies conducted both in vitro and in animal models indicated that the following possible mechanisms may explain hypoglycemic effects of Olea europaea: (1) a rise in the absorption of glucose in the peripheral tissue; (2) the augmentation of insulin sensitivity; (3) the promotion of insulin secretion; (4) the rise in the release of glucagon-like peptide-1 (GLP-1); (5) the blocking of α-glucosidase and α-amylase activity; and (6) the suppression of protein glycation and the reduction in advance glycation end-product (AGE) manufacture [170] (Figure 9).

#### 3.9.2. Clinical Trials

There are studies in the literature that have investigated the possible ability of *Olea europaea* to reduce blood glucose levels (Table 9).

In a fourteen-week trial, 79 patients with type 2 diabetes using oral hypoglycemic drugs and/or diet were given either 500 mg/day of OLE or a placebo. A decrease in FPI (*p* = 0.01) and HbA_1c_ (*p* = 0.037) was found compared to placebo, after supplementation [171].

One study was performed on forty-six overweight participants randomly assigned to receive four OLE capsules or a placebo daily for 3 months and then switched to the other treatment after a 6-week wash-out phase. The supplement contained 9.7 mg of hydroxytyrosol and 51.1 mg of oleuropein per daily dose. Comparing OLE supplementation to a placebo, it was found that both insulin sensitivity (*p* = 0.024) and pancreatic β cell function (*p* = 0.013) ameliorated. Furthermore, the supplement lowered PPG at 30 and 60 min (*p* = 0.008 and *p* = 0.005, respectively), PPI at 60 min (*p* = 0.004), as well as AUC of glucose (*p* = 0.008) and insulin (*p* = 0.041) in comparison to a placebo [172].

A sample of eleven overweight patients with T2DM, who were not receiving insulin treatment, drank 25 mL per day of refined olive oil (ROO) without measurable polyphenols for four weeks and high polyphenol extra-virgin olive oil (HP-EVOO) for an additional four weeks. After consuming HP-EVOO as opposed to ROO, a substantial drop in FPG (*p* = 0.023) and HbA_1c_ (*p* = 0.039) was noted [173].

In a different trial, fifty-seven prediabetic participants having FPG levels between 100 and 125 mg/dL and/or HbA_1c_ levels ranging from 5.7 to 6.4% ingested with meals 990 mL/day of olive leaf tea that included either 5 g (OLT) or 0.5 g of tea leaves (OLT at low concentration). After twelve weeks of supplementation, it was seen that, compared to OLT at low concentration, OLT considerably decreased FPG (*p* = 0.016) [174].

For three months, 148 subjects with IFG were daily given a standard diet along with or without 70 mL of an infusion of marigold and olive leaves (Olife^®^, Evergreen Life Products Srl., San Giovanni al Natisone, Italy) at breakfast. A significant reduction in FPG and PPG was observed compared to initial values (*p* < 0.05 for both) as well as to only the diet (*p* < 0.05 for both) and in HOMA index (*p* < 0.05) compared to baseline. Furthermore, compared to 4.1% of participants who received only the diet, 28.3% of patients with IFG who took the supplement became euglycemic when the study finished [175].

In combination with other nutraceuticals, *Olea europaea* has been shown in various human studies to have a hypoglycemic effect.

In the study by Said et al., 16 individuals with newly diagnosed type 2 diabetes were enrolled and placed under treatment with the diet, 11 of which had FPG levels below 300 mg/dL, whereas the remaining ones had levels equal or above 300 mg/dL. For four weeks, each participant took Glucolevel, a combination of dry leaf extracts from *Olea europea* L., *Juglans regia* L., *Urtica dioica* L. and *Atriplex halimus* L., at the dose of three tablets per day each containing 353 mg of dried herbs. During the first week of Glucolevel ingestion, there was a substantial decrease in FPG (*p* < 0.001) in all subjects compared to baseline readings. Levels of FPG clinically suitable were reached after two to three weeks of supplementation for participants with blood glucose levels below 300 mg/dL, and at the conclusion of treatment for those with blood glucose levels above 300 mg/dL. In addition, when compared to standard hypoglycemic drugs, the supplement substantially lowered HbA_1c_ (*p* < 0.05) in 6 diabetics in a test conducted on 22 patients with T2DM who were not completely responsive to traditional treatments and continued to have elevated FPG levels [176].

A supplement containing 1 g of OLE, 200 mg of green coffee bean extract, and 300 mg of beetroot powder, or a placebo, was given daily for six weeks to 37 patients with mildly elevated blood pressure who were not receiving treatment. Subsequently, they took it for another six weeks. The supplement did not change FPG, FPI, or HOMA index [177].

In a similar way, eighty hypercholesterolemic and overweight patients with low cardiovascular risk consumed a nutraceutical combination including olive fruit extract 25 mg, fermented red rice, sterol esters and stanols 720 mg, and curcumin 250 mg or a placebo per day for three months, along with diet and exercise. Compared to baseline and placebo, the supplement had no effect on FPG [178].

One trial was conducted on 663 subjects, including 134 prediabetics and 44 diabetics, who had blood pressure values borderline or Grade 1 hypertension not being treated or receiving medication. All participants were given Tensiofytol^®^, a supplement accessible on the market composed of dry extracts of leaves, 167 mg, and fruits, 53 mg, of *Olea europaea*, 2 capsules/day for 2 months. At the end of treatment FPG decreased in all subjects and much more in subjects affected by diabetes (*p* < 0.0001 for all) compared to baseline [179].

A proprietary mixture named TOTUM-63, which contains extracts from black pepper, bilberry, artichokes, olive leaf, and *Chrysanthellum*, was tested in fourteen male overweight patients who daily ingested TOTUM-63 2.5 g for four weeks followed by 5 g of supplement for an additional four weeks. The intervention phases were separated by two weeks of wash-out. In addition, the subjects undergone a carbohydrate tolerance test both before and after the maximum dosage was given. Following supplementation, there was a substantial decrease in PPG at 30 and 45 min (*p* < 0.05 for both) and glucose peak (*p* < 0.01) compared to before the consumption of TOTUM-63 at the highest dose. Furthermore, a considerable reduction in PPI at 45, 60, and 90 min, as well as in insulin peak, AUC, and insulin sensitivity index (*p* < 0.05 for all) was found, whereas the lowering in glucose AUC did not achieve statistical significance (*p* = 0.08) following supplementation [180].

One study examined how TOTUM-63 affected glycemic markers in 51 individuals with prediabetes or recently diagnosed type 2 diabetes who received 5 g of nutraceutical or placebo for daily six months. After supplementation, there was a substantial decrease in FPG and 2h-PPG (*p* < 0.05 for both) compared to placebo [181].

## 4. Supplements

### 4.1. Alpha-Lipoic Acid

Alpha-lipoic acid (ALA) is a natural occurring essential fatty acid that is synthesized in mitochondria from octanoic acid, but is also found in many foods and absorbed from many organs and tissues including bowel, liver, kidney, and brain [182].

Alpha-lipoic acid and its reduced form dihydro-lipoic acid (DHLA) are believed to have antioxidant activity in scavenging oxygen free radicals and restoring glutathione levels. Alpha-lipoic acid also acts as chelating metals and regenerates endogenous antioxidants such as vitamins C and E. In addition, ALA seems to have a potential role in the regulation of glucose metabolism and insulin activity, the decrease in lipid levels and the increase in nitric oxygen. Furthermore, in one study, it was reported that ALA improved peripheral diabetic polyneuropathy and other insulin resistance conditions, such as metabolic syndrome, PCOS, and obesity [183]. We focused on the potential glucose-lowering effects of ALA.

#### 4.1.1. Mechanisms of Action

**Figure 10:**
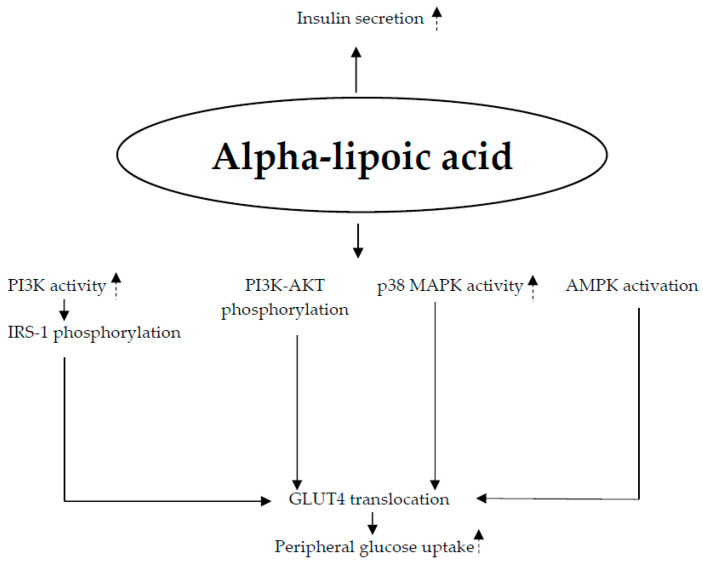
Possible mechanisms of action for alpha-lipoic acid in glycemic control. AKT: serine/threonine kinase; AMPK: adenosine monophosphate-activated protein kinase; GLUT4: glucose transporter; IRS-1: insulin receptor substrate type 1; MAPK: mitogen-activated protein kinase; PI3K: phosphatidylinositol 3-kinase. 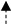: increase.

Many studies showed that both ALA and DHLA may increase glucose uptake by several molecular pathways. One of these involves the activation of insulin receptor cascade. Alpha-lipoic acid stimulates the activity of PI3K and the phosphorylation of IRS-1 leading to GLUT4 translocation and increased glucose uptake. Other mechanisms include the induction of PI3K and AKT phosphorylation, the increase in p38 mitogen-activated protein kinase (MAPK) activity or the activation of AMPK by ALA, which all lead to GLUT4 translocation. The effect of ALA on GLUT4 makes it an insulin mimetic agent. This substance also acts on the structure of pancreatic β cells protecting them from atrophy and increases insulin secretion [183] (Figure 10).

#### 4.1.2. Clinical Trials

Several studies investigated the hypoglycemic effects of ALA (Table 10).

One trial included 84 patients with type 1 or type 2 diabetes of which 35 received ALA 600 mg/day and the remaining served as a control group. After 18 months, no significant change was recorded in HbA_1c_ levels following supplementation [184].

In an open-label case-controlled study 12 patients with T2DM well controlled were enrolled and took 1200 mg/day of ALA for 4 weeks. Twelve subjects with normal glucose tolerance were used as a control group. The supplement significantly increased insulin sensitivity expressed as glucose metabolized (M) (*p* < 0.01) and ISI (*p* < 0.05) in diabetics compared to baseline values [185].

Lukaszuk reported that the consumption of R-ALA 200 mg thrice days 30 min before meals or placebo in 12 T2DM patients for 91 days determined a significant decrease more than 25% of HbA_1c_ values only in 2 subjects [186].

One trial included 57 T2DM who were given ALA 300 mg/day or placebo for 8 weeks. The trial found a significant reduction in FPG (*p* = 0.0001), PPG (*p* = 0.023), and HOMA index (*p* = 0.044) compared to baseline, as well as a significant reduction in FPG (*p* = 0.001) and HOMA index (*p* = 0.006) compared to placebo after supplementation. There was also a significant decrease in glutathione peroxidase (*p* = 0.035) compared to initial values [187].

In a 20-week trial, 360 obese subjects with hypertension, diabetes mellitus, or hypercholesterolemia were randomized to receive ALA 1200 or 1800 mg/day or placebo. Subjects with diabetes who consumed the supplement at the daily dose of 1800 mg showed a significant decrease in HbA_1c_ (*p* < 0.05) from baseline [188].

Another study investigated the effects of ALA consumption on glycemic parameters in 22 obese with IGT subjects of which 13 were treated with the supplement 600 mg in normal saline whereas 9 patients with only same amount of saline intravenously once daily for 2 weeks. The supplement significantly decreased 2h-PPG (*p* < 0.01) whereas increased M value and ISI (*p* < 0.01) compared to initial values [189].

Porasuphatana et al. evaluated the effects of ALA on glucose metabolism in 38 T2DM patients randomly assigned to receive placebo or various doses of supplement (300, 600, 900, and 1200 mg/day) for 6 months. It was found that FPG and HbA_1c_ were significantly decreased (*p* < 0.05 for both) after supplementation compared to placebo, and the reduction occurred in a dose-dependent manner [190].

Another trial enrolled 104 T2DM patients who were randomized into four groups to daily receive ALA 300 mg soft gelatin capsules, EPA 180 mg + DHA 120 mg six soft gelatin capsules, vitamin E 400 mg soft gelatin capsules or placebo, respectively, for three months. There was a significant decrease in HbA_1c_ after supplementation with ALA (*p* = 0.02), omega-3 (*p* = 0.003) and vitamin E (*p* = 0.009) compared to baseline [191].

In the study by Manning et al. including 160 subjects with metabolic syndrome who took ALA 600 mg/day, vitamin E 100 IU/day, ALA + vitamin E or placebo for 1 year, there was no significant change in FPG, insulin, and HOMA-IR after the administration of ALA alone or in combination [192].

Zhao and Hu evaluated the effect of ALA on glucose parameters in 90 aged T2DM patients with acute cerebral infarction who were given ALA 600 mg or vitamin C 3 g in 0.9% sodium chloride intravenously once daily for 3 weeks. At the end of treatment FPG, 2h-PPG, HbA_1c_, and HOMA-IR were significantly reduced, while HOMA-B was increased, compared to baseline (−*p* < 0.01 for all) and to control (*p* < 0.01 for all) [193].

A total of 60 obese T2DM patients with signs of peripheral polyneuropathia treated with metformin 850–1700 mg/day were divided in two groups of 30 subjects each. One group took ALA 600 mg/day for 20 weeks, whereas the other was used as a control group. The supplement caused a significant reduction in FPG (*p* < 0.001) compared to initial values and to control group [194].

One trail enrolled 71 adolescents with T1DM randomized into three groups: ALA 400 mg/day + antioxidant diet, placebo + antioxidant diet, and controls. The supplement significantly reduced daily insulin requirement (*p* = 0.049) and percentage of bolus dose (*p* = 0.047) compared to baseline after 3 months of treatment [195].

Another study showed that 40 T2DM patients with chronic periodontal disease who were given systemically ALA 1800 mg/day after scaling and root planning (SRP) or SRP alone for three months showed a significant decrease in HbA_1c_ (*p* < 0.001) compared both to baseline and to placebo [196].

In a randomized, double-blinded, placebo-controlled crossover study, 12 prediabetic and dyslipidemic subjects received ALA 600 mg/day or placebo for one month, separated by a wash-out period of 30 days. The supplement significantly reduced FPI (*p* = 0.07) and HOMA-IR (*p* = 0.04) compared to placebo [197].

In a retrospective, observational study, 322 patients were enrolled with euglycemia or dysglycemia who were given different dosages of ALA (400, 600, 800, and 1200 mg/day) over the last four years. A notable decrease in FPG was seen after supplementation with the dose of 800 and 1200 mg/day (*p* < 0.05 for the dose of 800 mg/day; *p* < 0.01 for the dose of 1200 mg/day) compared to baseline. In addition, ALA at 1200 mg/day significantly decreased FPG (*p* < 0.05) compared to the dose of 400 mg/day. Some dysglycemic patients treated with ALA at 800 and 1200 mg/day returned to euglycemic condition (5 subjects with IFG and 1 with IGT after the consumption of 800 mg/day; 11 subjects with IFG and 3 with IGT after the consumption of 1200 mg/day) [198].

The hypoglycemic effects of ALA in conjunction with other nutraceuticals were demonstrated in several clinical investigations.

In a randomized, double-blind, placebo-controlled trial, 102 TDM2 patients were divided into four groups to take ALA 600 mg, α-tocopherol 800 mg, α-tocopherol 800 mg + ALA 600 mg, or placebo, respectively, daily for 4 months. There were not significant improvements in FPG levels (*p* = 0.70) in the groups receiving ALA alone or and in combination with vitamin E, as well as in insulin (*p* = 0.54) and HOMA index (*p* = 0.77) in the ALA group [199].

Derosa et al. evaluated the hypoglycemic effects of a nutraceutical combination containing ALA 600 mg, L-carnosine 165 mg, zinc 7.5 mg, and vitamins B complex in 105 T2DM patients who were given the nutraceutical or placebo for three months. It was observed that the nutraceutical combination significantly reduced FPG, PPG, HbA_1c_, and HOMA-IR (*p* < 0.05 for all) compared both to baseline and to placebo. A significant increase in superoxide dismutase and glutathione peroxidase, as well as a decrease in malondialdehyde (*p* < 0.05 for all), was also found compared to initial values and to placebo [200].

One study showed the effect of a nutraceutical combination containing ALA, berberine, *Lagerstroemia speciosa*, curcumin, chromium picolinate, and folic acid on glucose homeostasis already previously cited [27].

A total of 82 obese T2DM patients were randomized to receive a supplement consisting of 7 mg ALA/kg body weight, 6 mg carnosine/kg body weight, and 1 mg thiamine/kg body weight 3 times/day or placebo for 8 weeks. At the end of treatment, FPG and HbA_1c_ were significantly reduced (*p* < 0.01 for FPG; *p* < 0.05 for HbA_1c_), whereas insulin increased (*p* < 0.05) compared to baseline after supplementation. Moreover, HOMA-IR was increased (*p* < 0.05) as confirmed by the reduction in the QUICKI and HOMA-B (*p* < 0.05 for both) in the supplementation group compared to initial values [201].

One study investigated the effects of the association of ALA, *Vitis vinifera* L., and *Ginkgo biloba* (Blunorm forte^®^, Laborest s.r.l., Assago, Italy) on glycemic markers in 123 T2DM patients with erectile dysfunction. The patients were divided in four groups who received the following for 3 months: placebo, 1 tablet/day, 40 min before breakfast and 15–30 min before sexual act; placebo, 1 tablet/day, 40 min before breakfast and Avanafil, a selective inhibitor of phosphodiesterase type 5 used for the treatment of erectile dysfunction, 200 mg, 15–30 min before sexual act; Blunorm forte^®^, 1 tablet/day, (ALA, 400 mg; *Vitis vinifera* L., 200 mg; and *Ginkgo biloba*, 80 mg) 40 min before breakfast and Avanafil, 200 mg, 15–30 min before sexual act; and Blunorm forte^®^, 2 tablets/day, 40 min before breakfast and dinner and placebo, 1 tablet, 15–30 min before sexual act. The supplement alone determined a substantial reduction in FPG and HOMA-IR (*p* < 0.05 for both) compared to baseline and to placebo, as well as in association with Avanafil (*p* < 0.05 for both) compared both to initial values and to placebo [202].

### 4.2. Omega-3

Bioactive substances known as omega-3 polyunsaturated fatty acids (*n*-3 PUFAs) include eicosapentaenoic acid (EPA, 20:5*n*-3) and docosahexaenoic acid (DHA, 22:6*n*-3) obtained mainly from fish and shellfish, and alpha-linolenic acid (ALA, 18:3*n*-3) derived from green leafy vegetables, seeds, and nuts. It has been hypothesized that supplementation with *n*-3 PUFAs could improve glycemic parameters [203].

#### 4.2.1. Mechanisms of Action

**Figure 11:**
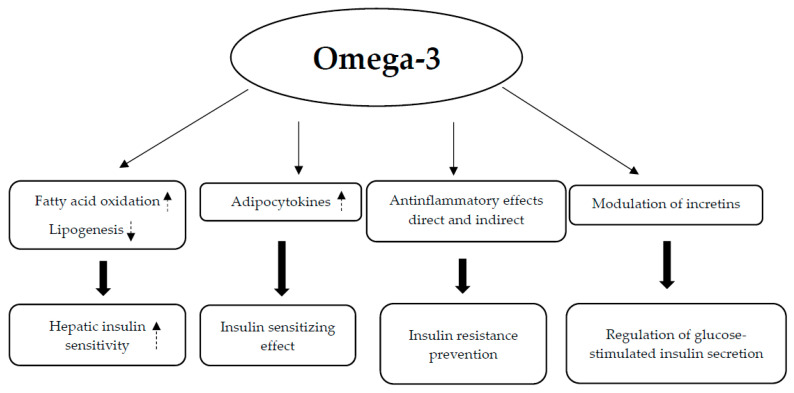
Potential hypoglycemic mechanisms of action of Omega-3. 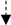: decrease; 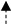: increase.

Although the mechanisms through which *n*-3 polyunsaturated fatty acids improve glycemic control are not currently well understood [204], in vivo studies on animal models described some potential processes including (a) enhancement of insulin sensitivity in the liver [205] through lowered lipogenesis and increased fatty acid oxidation [206,207], (b) insulin sensitizing effect through the increase adipocytokines release [208] and (c) avoidance of insulin resistance by both direct [209] and indirect [210,211] anti-inflammatory actions. Furthermore, incretins implicated in glucose-stimulated insulin release can be modulated by *n*-3 PUFAs [212] (Figure 11).

#### 4.2.2. Clinical Trials

Clinical studies that have been published in the literature have produced conflicting results about how *n*-3 PUFAs affect glucose metabolism (Table 11).

Early human research revealed that giving this supplement to patients with type 2 diabetes did not enhance glucose metabolism.

In this context, it was found that six men with type 2 diabetes non-insulin-dependent who received daily treatment with 18 g fish oil concentrate containing 5.5 g of *n*-3 PUFAs (3.3 g EPA and 2.2 g DHA) and 108 mg of cholesterol for one month experienced a rise in FPG and HbA_1c_ (*p* = 0.03 for both). Furthermore, it was shown that FPI decreased marginally but not significantly (*p* = 0.22) from baseline, and, after withdrawal from the supplement, FPG reverted to baseline values [213].

In a different study, eight men with T2DM non-insulin-dependent who were given 8 g of *n*-3 PUFAs per day for eight weeks exhibited a significant increase in FPG (*p* = 0.005) whereas HbA_1c_ and FPI remained unchanged [214].

According to Borkman et al., when 10 patients with T2DM non-insulin-dependent ingested 10 g of fish oil (18% EPA and 12% DHA) daily for three weeks, FPG levels significantly increased (*p* < 0.05) without affecting their FPI and C-peptide compared to initial values obtained from subjects receiving only diet. Moreover, following a three-week wash-out period, FPG levels were observed to revert to baseline values [215].

On the contrary, one trial showed that after 10 weeks of treatment with 2.7 g of *n*-3 PUFAs (1.8 g EPA and 0.9 g DHA) daily, 12 individuals with type 1 diabetes did not show changes in FPG and HbA1c [216].

However, FPG levels were found to rise after 3 and 6 weeks in a study which involved 80 patients with type 2 diabetes non-insulin-dependent received 10 g per day of fish oil (1.8 g EPA and 1.2 g DHA) for 6 weeks. This increase was significant only after 3 weeks of supplementation (*p* = 0.01) compared to control, while HbA_1c_ did not change [217].

According to Rillaerts et al., this supplement had no effect on glycemic control in further research. At this regard, the administration of *n*-3 PUFAs 3.1 g per day for three weeks to 20 obese subjects with type 2 diabetes who were not insulin-dependent did not change FPG, PPG, or HbA_1c_ values [218].

Similiarly, in a sample of 40 patients with type 2 diabetes non-insulin-dependent and hyperlipidemia who were given 9 and 18 g per day of fish oil (EPA 28.8% and DHA 27.3%) for 12 weeks, the supplement did not alter FPG and HbA_1c_ [219].

Another study investigated the effect of omega-3 fatty acid consumption on glucose parameters in 28 patients with T2DM who are not insulin-dependent assigned to a higher or lower ratio of dietary polyunsaturated to saturated fatty acid (P/S). All subjects, initially, received capsules of olive oil as placebo at a dose equivalent to 35 mg of oleic acid (18:1) per kg body weight per day for 3 months. Subsequently they were given a linseed oil equivalent to 35 mg of 18:3 omega-3 per kg body weight per day or a fish oil equivalent to 35 mg of 20:5 omega-3 + 22:6 omega-3 per kg body weight per day. Each supplementation was consumed for 3 months. At the end of each 3-month intervention a 7-day diet record was completed to calculate the dietary P/S ratio and dietary nutrient intake. Fasting glucose, insulin, glucagon and C-peptide levels were not modified by either omega-3 fatty acid. However, these parameters resulted lower in patients who were given the high P/S diet [220].

The results of Sirtori et al. indicated that there were no differences in HbA_1c_, FPG, or FPI levels between 414 patients with type 2 diabetes non-insulin-dependent who received *n*-3 PUFAs 2.6 g (1530 mg EPA and 1050 mg DHA) daily for two months and 1.7 g (1020 mg EPA and 700 mg DHA) daily for four months. The glycemic profile did not worsen after a year of supplementation, as shown by the assumption of *n*-3 PUFAs at 1.7 g per day for an extra six months [221].

Another study found that in twenty-six postmenopausal women with type 2 diabetes, the daily administration of 3 g of fish oil (1.08 g EPA and 0.72 g DHA) for 2 months did not affect the concentrations of FPG, FPI, and HbA_1c_, nor the values of HOMA-B and the homeostatic model assessment of insulin sensitivity (HOMA-IS) [222].

Derosa et al. showed that, in 333 individuals with mixed dyslipidemia, daily supplementation with 3 g of *n*-3 PUFAs (0.9–1.5 proportion of EPA and DHA) for six months did not alter FPG, FPI, and HOMA-IR compared to starting values [223].

According to Wong et al., after 12 weeks of supplementation, 97 patients with type 2 diabetes who took 4 g per day of fish oil (EPA 42% and DHA 25%) did not exhibit changes in FPG and HbA_1c_ [224].

As previously mentioned, a 6-month study involving 124 elderly patients who took fish oil in the form of capsule 1g per day containing 300 mg *n*-3 PUFAs (180 mg EPA and 120 mg DHA) demonstrated that FPG, FPI and HOMA-IR did not modify after the supplementation in comparison to baseline and placebo [225].

In one hundred eleven hypertriglyceridemic individuals with normal-high blood pressure that does not receive treatment, a retrospective study found that *n*-3 PUFAs (85% of EPA and DHA in the ratio 0.9–1.5) 2 g per day was effective. There was no variation in FPG levels during a 12-month treatment period, with the exception of a significant rise after three months (*p* < 0.01) compared to starting values [226].

Conversely, 167 patients with combined dyslipidemia who received *n*-3 PUFAs (EPA 1200 mg and DHA 1350 mg) 3 g daily for six months experienced a substantial reduction in FPG (*p* < 0.05) compared to initial values, while FPI and HOMA-IR remained unchanged. The supplement also raised the total glucose requirement (TGR) and M value (*p* < 0.05 for both) in individuals receiving euglycemic hyperinsulinemic clamp in comparison to baseline [227,228].

Nevertheless, 12.536 subjects with dysglycemia at high risk of cardiovascular events who were given *n*-3 PUFAs (EPA 465 mg and DHA 375 mg) 1g per day, did not exhibit differences in FPG and HbA_1c_ compared to placebo during a median 6.2-year follow-up period [229].

A sample of forty-one women with hypertension and type 2 diabetes took fish oil at the daily dose of 2.5 g (EPA 547.5 mg and DHA 352.5 mg), 1.5 g (EPA 328.5 mg and DHA 211.5 mg) or placebo. Following one month of treatment, the lowest *n*-3 PUFAs dose determined the greater frequency of FPG (42.9%) and HbA1c (35.7%) decrease, without reach statistical significance, compared to other treatments. In contrast, 85.7% of females supplemented with the highest dose of *n*-3 PUFAs showed a lowered of QUICKI, a marker of insulin sensitivity as well as 35.7% of women receiving the lowest dose of supplement exhibited a decrease in HOMA-IR similarly to those given placebo (38.5%) [230].

Afterwards, it was shown that fish oil (EPA 400 mg and DHA 1450 mg) at 2.4 g per day, given for eight weeks to 72 patients with type 2 diabetes, had no effect on HOMA-IR values, FPG levels, or FPI levels compared to placebo [231].

In the study by Derosa et al., it was demonstrated that *n*-3 PUFAs can also enhance glucose metabolism [227,228]. The same authors assessed the hypoglycemic effect of this supplement, 3 g per day (EPA and DHA in the ratio of 0.9–1.5) in two hundred eighty-one overweight/obese subjects with IFG or IGT during an 18-month trial. At 9 months and at the treatment’s conclusion, there was a significant reduction in FPG and HOMA-IR compared to baseline (at 9 months: *p* < 0.05 for both; at 18 months: *p* < 0.01 for FPG and *p* < 0.05 for HOMA-IR) and to placebo (at 9 months: *p* < 0.05 for both; at 18 months: *p* < 0.01 for both). In addition, the supplement decreased FPI levels at 9 months (*p* < 0.05) and 18 months (*p* < 0.01) compared to baseline and at the end of therapy (*p* < 0.05) compared to placebo [232].

A total of 107 newly diagnosed impaired glucose metabolism (IGM) patients with coronary artery disease (CAD) were given either EPA at the dose of 1800 mg/day or no EPA for 6 months. Fasting plasma glucose, HbA_1c_, and HOMA-IR were unaffected by the supplement. However, EPA determined a significant reduction in glucose AUC and incremental glucose peak (*p* < 0.0001 for both), as well as a mild not significant increase in immune reactive insulin AUC (*p* = 0.08) compared to baseline. In addition, immune reactive insulin AUC/glucose AUC ratio, an index of postprandial insulin secretory ability, was significantly increased (*p* < 0.0001) compared to initial values after supplementation [233].

Moreover, Jacobo-Cejudo et al. demonstrated that giving 54 patients with T2DM *n*-3 PUFAs 520 mg (EPA 320 mg and DHA 200 mg) for six months resulted in a reduction in FPG (*p* = 0.011) and HbA_1c_ (*p* = 0.009) compared to initial values. In addition, FPI and HOMA-IR were significantly raised (*p* = 0.0001 for both) by the supplement compared to values before the *n*-3 PUFAs consumption [234].

About the ability of *n*-3 PUFAs to reduce blood sugar when used with other nutraceuticals, the effects of combination fish oil capsules with curcumin tablets have been previously shown [235].

### 4.3. Essential Amino Acids

Essential amino acids are a class of amino acids that cannot be synthesized from scratch by the organism fast enough to supply their demand and they must necessarily come from the diet. This class includes histidine, isoleucine, leucine, lysine, methionine, phenylalanine, threonine, tryptophan, and valine. Leucine, isoleucine and valine are characterized by the presence of a lateral branched-chain and are also called branched-chain amino acid (BCAA) [236].

There is another class of amino acids defined semi-essential including arginine, cysteine, and tyrosine that can be synthesized endogenously, but in insufficient amounts, and then are required partially to be obtained from the diet [236].

Recent metabolomics-based studies showed perturbation of normal amino acid metabolism in obese, insulin-resistant and T2DM subjects who presented an increase in blood and urine concentrations of specific amino acid classes [237].

Alterations in the metabolism of these amino acids are closely involved in the pathogenesis of T2DM and are associated with the onset of cardiovascular disease. Therefore, these perturbed amino acids could be investigated as therapeutic targets or corrected through supplementation in order to ameliorate T2DM management and prevent associated cardiovascular complications [236].

#### 4.3.1. Mechanisms of Action

Essential amino acids are thought to modulate glucose homeostasis through insulinotropic and non-insulinotropic mechanisms. As regards the insulinotropic activity of essential amino acids, this can be exerted through direct and indirect modulation of pancreatic β-cell function and, to a lesser extent, α-cell function. One of the most established direct insulinotropic mechanisms is known as the “triggering pathway”, comprising ATP production from the tricarboxylic acid cycle, which requires oxidized amino acid substrates. Adenosine triphosphate, in turn, determined the closure of ATP-sensitive potassium channels producing β-cell membrane depolarization, which leads to the activation of calcium channels with intracellular calcium influx and then insulin exocytosis. Another direct insulinotropic mechanism consists of the activation of the mammalian target of rapamycin complex 1 (mTORC1) in β cells by essential amino acids, resulting in insulin synthesis [238].

The indirect insulinotropic mechanisms involve the incretins, glucose-dependent insulinotropic peptide (GIP), and GLP-1, released from enteroendocrine cells, which promote insulin release and dampen glucagon secretion. In addition, GLP-1 stimulates β-cell proliferation and survival, and it also reduces their apoptosis. Essential amino acids may also indirectly regulate insulin and glucagon release through the vagus nerve that facilitates communication within the gut–brain axis via its afferent and efferent branches. Vagal afferent neurons that innervate the lamina propria of the bowel mucosa send sensory information about nutrient and peptide hormone concentrations to the dorsal vagal complex of the brainstem, whereas the vagal efferent ones transmit motor information to the endocrine pancreas, regulating insulin and glucagon release [238].

**Figure 12:**
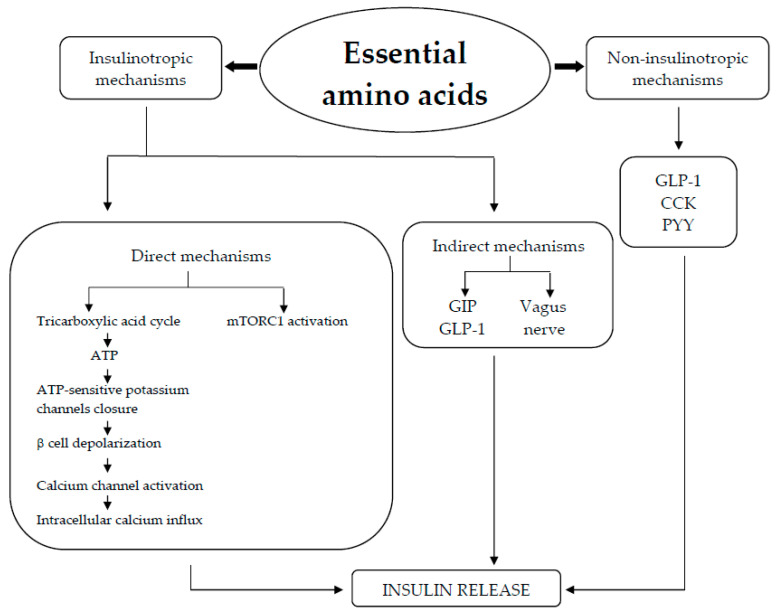
Possible mechanisms of action for essential amino acids in glycemic homeostasis. ATP: adenosine triphosphate; CCK: cholecystokinin; GIP: glucose-dependent insulinotropic peptide; GLP-1: glucagon-like peptide-1; PYY: peptide tyrosine–tyrosine; mTORC1: mammalian target of rapamycin complex 1.

The non-insulinotropic mechanisms include GLP-1, already known for its insulinotropic properties, and the anorectic hormones cholecystokinin (CCK) and peptide tyrosine–tyrosine (PYY) that regulate food intake. These hormones are secreted into the bowel in the presence of essential amino acids following meal consumption and can inhibit gastric motility and emptying, as well as reduce gastric acid secretion. It is believed that GLP-1, CCK, and PYY directly modulate the vagal and enteric nervous systems, which innervate enteroendocrine cells [238] (Figure 12).

#### 4.3.2. Clinical Trials

Many studies found in the literature showed contrasting results regarding the hypoglycemic effects of essential amino acids (Table 12).

One study assessed how L-tryptophan affected plasma glucose levels in 21 young healthy subjects who received 10 g of this amino acid per os or placebo. It was observed that blood sugar levels reached their highest value (*p* = 0.02) 180 min after L-tryptophan intake, as well as insulin (*p* = 0.002) at 140 min, compared to basal values [239].

Another trial enrolled 43 healthy subjects who were administered the following meals on separate days: (1) control (C) (480 mL of a study beverage containing carbohydrate 82 g, protein 20 g and fat 14 g; (2) control plus phenylalanine 3.5 g and leucine 3.5 g (AA); (3) control plus 1 g of Salacia oblonga extract, an herbal α-glucosidase inhibitor (S); and (4) control plus *Salacia oblonga* extract 1 g, phenylalanine 3.5 g and leucine 3.5 g (SAA). Glucose AUC values, from 0 to 120 min, were significantly decreased for the S (*p* = 0.035) and SAA (*p* = 0.046) treatments, whereas those of glucose AUC, from 0 to 180 min, were reduced only for SSA (*p* = 0.039) compared to control. Insulin AUC data, from 0 to 120 min, were decreased for S compared to control (*p* < 0.001), AA (*p* < 0.001), and SAA (*p* = 0.003). Similarly, insulin AUC values, from 0 to 180 min, were reduced for S compared to control, AA, and SAA (*p* < 0.001 for all) [240].

In an 18-month study, 144 subjects with IGT and metabolic syndrome who received L-arginine 6.4 g/day or placebo were enrolled. The essential amino acid significantly improved insulin levels at 120 min after OGTT (*p* = 0.01), proinsulin/c-peptide ratio (*p* = 0.01), and insulinogenic index (IGI)/HOMA-IR (*p* = 0.04) compared to placebo. At the end of treatment, 42.40% of subjects receiving L-arginine, compared to 22.10% of the ones given placebo, returned to euglycemia (*p* = 0.001). After the 12-month extended follow-up period, it was reported that insulin levels at 120 min after OGTT (*p* < 0.05), proinsulin/c-peptide ratio (*p* = 0.01), and IGI/HOMA-IR (*p* < 0.05) remained significantly ameliorated in the L-arginine group compared to the placebo group. The essential amino acid also significantly decreases 2h-PPG (*p* = 0.03) and HbA_1c_ (*p* < 0.05) compared to placebo. In addition, in subjects previously treated with L-arginine, the cumulative incidence of diabetes was decreased (*p* < 0.05), whereas the cumulative probability to return euglycemic increased compared to placebo (*p* < 0.001) [241]. After a 90-month extended follow-up period, it was shown that the cumulative incidence of diabetes was 40.60% in the L-arginine group and 57.40% in the placebo group (*p* < 0.02). In individuals that remained free of diabetes, the essential amino acid improved proinsulin/c-peptide ratio (*p* < 0.001) and IGI/HOMA-IR (*p* < 0.01) compared to the placebo [241,242].

Steinert et al. investigated the effect of L-tryptophan on glycemic parameters in 10 healthy normal-weight men on three occasions in which they were given a 90 min intraduodenal infusion of the essential amino acid at 0.075 (L-Trp-0.075) or 0.15 kcal/min (L-Trp-0.15) or saline as control and then an ad libitum test meal. L-tryptophan-0.15 slightly increased insulin 75 min after infusion (*p* < 0.05) compared to control, whereas neither of the doses of the essential amino acid affected blood glucose [243].

The effects of leucine on glucose homeostasis were also assessed by the same researchers in 12 lean men who received 90 min intraduodenal infusions of this amino acid at 0.15 kcal/min (leucine-0.15) or 0.45 kcal/min (leucine-0.45) or control on three occasions, followed by an ad libitum test meal. The highest dose of leucine determined a significant decrease in blood glucose (*p* < 0.05) between 75 and 90 min, whereas both doses of this amino acid increased insulin (*p* < 0.05) (leucine-0.45 between 15 and 90 min; leucine-0.15 between 15 and 30 min) compared to control. After the test meal, all treatments increased blood glucose and insulin (*p* < 0.001) [244].

Two separate randomized crossover studies both enrolled 12 healthy subjects who took, on three separate visits, an intragastric infusion of leucine 5 g or 10 g or control, and isoleucine 5 g or 10 g or control, respectively. All volunteers consumed a mixed-nutrient drink 15 min later. It was observed that leucine at 10 g significantly reduced glucose AUC (*p* < 0.05), tended to reduce peak glucose (*p* = 0.072), and increased insulin and C-peptide AUCs (*p* < 0.01); and isoleucine at 10 g decreased glucose AUC (*p* < 0.01) and the peak glucose (*p* < 0.01) compared to control [245].

Ullrich et al. reported the effects of L-lysine on glycemic parameters in 12 healthy volunteers who were administered intragastric infusions containing 5 g or 10 g of this amino acid or a control solution on three occasions. For the next 15 min, the volunteers consumed a mixed-nutrient drink, and for the next hour (t = 0–60 min), blood samples were collected at 15-min intervals. The subjects also consumed a standardized ad libitum buffet meal at 60 min for ≤30 min (t = 60–90 min). It was observed that the two L-lysine doses did not differ in terms of effects on blood glucose and insulin. The amino acid did not affect blood glucose and insulin at 15 min or blood glucose and insulin AUC from 0 to 60 min compared to control. In response to the mixed-nutrient drink, L-lysine significantly reduced blood glucose (*p* < 0.01) and insulin (*p* < 0.05) at 60 min compared to control. In addition, blood glucose and insulin levels at 90 min were significantly lower after L-lysine than after control (*p* < 0.05 for blood glucose and *p* < 0.01 for insulin) [246].

One trial reported the effects of essential amino acids supplementation 15 g/day or placebo both alone and in association with aerobic exercise in 42 healthy older adults for 22 weeks. The supplement contained 40% L-leucine, 16.7% L-lysine, 11% L-valine, 10.7% L-isoleucine, 9.3% L-threonine, 6.7% L-phenylalanine, 3.3% L-methionine, 1.7% histidine, and 0.7% L-tryptophan. Essential amino acids alone and in combination with exercise did not change insulin sensitivity [247].

An acute, randomized, double-blind, placebo-controlled crossover study investigated the efficacy of oral 10 g L-phenylalanine on gastroenteropancreatic hormones release and glucose levels in 11 healthy subjects who received the essential amino acid, D-phenylalanine or placebo. The participants were also provided with an ad libitum test meal 70 min after treatment intake. A previous dose-finding study investigated the tolerability of escalating amounts of oral L-phenylalanine (0, 3, 6, and 10 g). The essential amino acid significantly increased insulin at 45, 60, and 70 min compared to D-phenylalanine and at 60 min compared to placebo prior to meal ingestion (*p* < 0.05 for all); the study also determined a significant increase in insulin AUC compared to D-phenylalanine (*p* = 0.0216) after meal intake. In addition, glucagon was increased by L-phenylalanine at 60 min compared to D-phenylalanine (*p* < 0.05) and at 70 min both to the essential amino acid (*p* < 0.05) and placebo (*p* < 0.01) in pre-prandial period as well as at 105 min compared to placebo (*p* < 0.05) and at 120 min compared to the essential amino acid (*p* < 0.05) and placebo (*p* < 0.01) in post-prandial period. L-phenylalanine also increased GIP levels relative to D-phenylalanine (*p* = 0.042) and placebo (*p* = 0.0249) 70 min following ingestion. Moreover, L-phenylalanine significantly decreased PPG AUC (*p* = 0.0317) compared to placebo [248].

Another study evaluated the hypoglycemic effects of leucine, isoleucine and valine in 15 healthy who were given, on four separate occasions, intragastrically 10 g of each amino acid or control 30 min before a mixed-nutrient drink. After the drink, leucine and isoleucine decreased peak glucose compared to control (*p* < 0.05 for both) and valine (*p* < 0.05 for both). In addition, isoleucine reduced glucose AUC_15–120min_ compared to control (*p* < 0.05) and valine (*p* < 0.05) [249].

The same authors investigated the hypoglycemic effects of leucine and isoleucine in 14 T2DM patients who received, on three separate visits, intragastric administration of each amino acid 10 g or control 30 min before a mixed-nutrient drink. It was shown that leucine and isoleucine did not affect glucose AUC or peak glucose levels. However, the amino acids increased insulin AUC before the drink (*p* < 0.001 for leucine and *p* = 0.008 for isoleucine) and after the drink (*p* = 0.021 for leucine and *p* = 0.034 for isoleucine) compared to control. It was also observed that leucine resulted in a rise of peak insulin concentrations (*p* < 0.05), whereas isoleucine increased glucagon AUC before (*p* = 0.048) and after drink (*p* = 0.031) when compared to control [250].

Alqudah et al. investigated the amino acids profile of 124 patients with type 2 diabetes using metformin and 67 healthy controls and the correlation with glycemic parameters. Regarding essential amino acids, there was found to be a significant increase in leucine (*p* < 0.01), lysine (*p* < 0.001), phenylalanine (*p* < 0.01) and tryptophan (*p* < 0.05) in T2DM patients compared to controls. These four amino acids were also positively correlated with FPG (leucine: r = 0.53, *p* = 0.004; lysine: r = 0.395, *p* = 0.03; phenylalanine: r = 0.42, *p* = 0.006; tryptophan: r = 0.675, *p* = 0.0001) and HbA_1c_ (leucine: r = 0.44, *p* = 0.02; lysine: r = 0.43, *p* = 0.012; phenylalanine: r = 0.49, *p* = 0.001; tryptophan: r = 0.59, *p* = 0.001) in diabetics indicating that alterations in the metabolism of these amino acids are closely involved in the pathogenesis of T2DM [236].

A total of 12 men with T2DM received, on three separate occasions, intragastric administration of 3 g tryptophan, 1.5 g tryptophan or control (0.9% saline) 30 min before a mixed-nutrient drink to assess glucose parameters. The both doses of tryptophan alone did not affect FPG, whereas there was a significant increase in C-peptide after 3 g and 1.5 g of the essential amino acid (*p* < 0.05) and C-peptide AUC after 1.5 g tryptophan (*p* = 0.002) compared to control. After mixed-nutrient drink the higher dose of the essential amino acid significantly reduced PPG from 15 to 30 min (*p* < 0.05) compared to control, as well as from 30 to 45 min (*p* < 0.05) compared to 1.5 g tryptophan. Following mixed-nutrient drink also C-peptide AUC tended to be greater after 1.5 g of the essential amino acid (*p* = 0.06) compared to control [251].

One study enrolled 36 elderly subjects with T2DM who were given 8 g of BCAA (4 g leucine, 2 g valine, and 2 g isoleucine) or 7.5 g of soy protein for 24 weeks. Supplementation did not alter FPG, FPI, HbA_1c_, and HOMA-IR over time in either group, and there were no differences noted between groups [252].

## 5. Conclusions

Evidence from clinical studies seems to indicate a role in the management of prediabetes for *Berberis, Banaba*, and *Gymnema sylvestre*. In contrast, clinical studies on *Ilex paraguariensis*, *Morus*, Omega-3, and Essential amino acids showed conflicting data on their glucose-lowering effects; however, well-designed long-term studies are necessary to corroborate these results. Further trials are also required for *Cinnamomum* and *Momordica charantia. Olea europaea* seems very interesting when used in prediabetes status, and *Ascophyllum nodosum* and *Fucus vesiculosus* also demonstrated hypoglycemic properties when combined. *Berberis*, *Ascophyllum nodosum*, and *Fucus vesiculosus* are also interesting in diabetes, but further and longer studies are needed. Moreover, alpha-lipoic acid could play an important role in the prevention and management of diabetes complications both for its glucose-lowering effects and its antioxidant and anti-inflammatory actions. However, further studies are necessary to consolidate the beneficial effects of alpha-lipoic acid on diabetes treatment.

## Figures and Tables

**Table 1:** Overview of clinical trials on glucose-lowering effects of *Ascophyllum nodosum* and *Fucus vesiculosus*.

First Author and Year	Participants	Therapy and Duration	Findings
Hall, A.C. (2012) [12]	Overweight or obesity patients (n = 12)	100 g of bread with *Ascophyllum nodosum* 4% 4 h following supplement intake	PPG no decrease
Iacoviello, L. (2013) [13]	Healthy subjects (n = 43)	2 capsules/day *Ascophyllum nodosum* 900 mg and iodine 175 μg 6 months	FPG, FPI, and HOMA index no effect
Murray, M. (2018) [14]	Healthy subjects (n = 38)	500 mg *Fucus vesiculosus* 2 g *Fucus vesiculosus* 2 h after carbohydrate consumption	PPG and PPI no effect Different insulin sensitivity in Asian subjects
Murray, M. (2019) [11]	Normotensive subjects (n = 18)	2 g/day *Fucus vesiculosus* 3 h after supplement ingestion	PPG no effects except reduction in females PPI iAUC and peak plasma insulin increase in Asian subjects
Paradis, M.E. (2011) [15]	Healthy subjects (n = 23)	500 mg *Ascophyllum nodosum* and *Fucus vesiculosus* 3 h after carbohydrate ingestion	No effect on glucose response Insulin iAUC decrease Cederholm index of insulin sensitivity increase
De Martin, S. (2018) [16]	Overweight or obesity patients (n = 50)	3 capsules/day *Ascophyllum nodosum* 237.5 mg, *Fucus vesiculosus* 12.5 mg, and chromium picolinate 7.5 μg 6 months	FPG, FPI, and HOMA index decrease
Derosa, G. (2019) [17]	Dysglycemic patients (n = 65)	3 capsules/day *Ascophyllum nodosum* 237.5 mg, *Fucus vesiculosus* 12.5 mg, and chromium picolinate 7.5 μg 6 months	FPG, PPG, HbA_1c_, and HOMA index decrease
Derosa, G. (2019) [18]	T2DM patients (n = 175)	3 capsules/day *Ascophyllum nodosum* 237.5 mg, *Fucus vesiculosus* 12.5 mg, and chromium picolinate 7.5 μg 6 months	FPG, PPG, and HbA_1c_ decrease

iAUC: incremental area under curve; FPG: fasting plasma glucose; FPI: fasting plasma insulin; HbA_1c_: glycated hemoglobin; HOMA index: homeostatic model assessment index; PPG: post-prandial plasma glucose; PPI: post-prandial plasma insulin; T2DM: type 2 diabetes mellitus.

**Table 2:** Recapitulation of human studies on hypoglycemic activity of *Lagerstroemia speciose*.

First Author and Year	Participants	Therapy and Duration	Findings
Ikeda, Y. (2002) [21]	IFG patients (n = 15)	100 mg/day banaba extract 1 year	FPG decrease
Judy, W.V. (2003) [19]	T2DM patients, non-insulin-dependent (n = 10)	16 mg/day; 32 mg/day; 48 mg/day banaba extract standardized to 1% corosolic acid 2 weeks	FPG decrease at the highest dose
Tsuchibe, S. (2006) [22]	IFG patients (n = 12)	10 mg/day corosolic acid 2 weeks	FPG and 1h-PPG decrease
Fukushima, M. (2006) [23]	IFG, IGT, and T2DM patients, subjects with normal glucose tolerance (n = 31)	10 mg corosolic acid 5 min before OGTT Different occasions	PPG from 60 to 120 min decrease
Choi, S. (2014) [24]	Prediabetes patients (n = 45)	300 mg/day banaba extract 2 g/day soybean leaf extract 12 weeks	FPG, HbA_1c_, and HOMA-IR decrease
Ikeda, Y. (1999) [25]	T2DM patients (n = 24)	9 tablets/day containing banaba extract, green tea, green coffee, *Garcinia cambogia*	FPG decrease
Kim, H.J. (2012) [26]	IGT and T2DM patients (n = 62)	6 g/day mixture of banaba leaf extract, mulberry leaf extract, Korean red ginseng powder 6 months	Glucose AUC decrease Downward trend insulin AUC
Cicero, A.F.G. (2017) [27]	IFG patients (n = 40)	500 mg/day *Lagerstroemia speciosa* + 310 mg/day berberine + 250 mg/day curcumin + 2.6 μg/day chromium picolinate + 0.30 mg/day folic acid + 220 mg/day α-lipoic acid 8 weeks	FPG, FPI, and HOMA index decrease
Derosa, G. (2020) [28]	IFG and IGT patients (n = 148)	40 mg/day banaba + 200 mg/day berberine + 200 mg/day curcumin + 300 mg/day inositol + 100 μg/day chromium picolinate 3 months	FPG, PPG, HbA_1c_, and HOMA-IR decrease FPI increase

AUC: area under the curve; FPG: fasting plasma glucose; FPI: fasting plasma insulin; HbA_1c_: glycated hemoglobin; HOMA-IR: homeostatic model assessment of insulin resistance; HOMA index: homeostatic model assessment index; IFG: impaired fasting glucose; IGT: impaired glucose tolerance; OGTT: oral glucose tolerance test; PPG: post-prandial glucose; T2DM: type 2 diabetes mellitus.

**Table 4:** Recapitulation of clinical trials on glucose-lowering effects of *Cinnamomum*.

First Author and Year	Participants	Therapy and Duration	Findings
Khan, A. (2003) [58]	T2DM patients (n = 60)	1 g/day, 3 g/day, or 6 g/day of *Cinnamomum cassia* 40 days	FPG decrease
Mang, B. (2006) [59]	T2DM patients (n = 79)	3 g/day of *Cinnamomum cassia* powder 4 months	FPG decrease
Vanschoonbeek, K. (2006) [60]	T2DM postmenopausal patients (n = 25)	1.5 g/day of *Cinnamomum cassia* 6 weeks	FPG, FPI, HbA_1c_, HOMA-IR, ISIcomp, and OGIS no decrease
Wang, J.G. (2007) [61]	Women with PCOS (n = 15)	1 g/day cinnamon extract 8 weeks	FPG and HOMA-IR decrease QUICKI and Matsuda insulin resistance index increase
Solomon, T.P. (2007) [62]	Lean, healthy volunteers (n = 7)	5 g of *Cinnamomum cassia* 2 h	AUC glucose decrease; Matsuda insulin resistance index increase
Blevins, S.M. (2007) [63]	T2DM patients (n = 60)	1 g/day of *Cinnamomum cassia* 3 months	FPG, FPI, and HbA_1c_ no decrease
Altschuler, J.A. (2007) [64]	Adolescent with T1DM (n = 72)	1 g/day cinnamon 3 months	No significant difference in HbA_1c_ total daily use of insulin and number of hypoglycemic episodes
Solomon, T.P. (2009) [65]	Healthy volunteers (n = 8)	3 g/day *Cinnamomum cassia* 14 days	PPG, PPI, AUC glucose and AUC insulin decrease Insulin sensitivity improve
Crawford, P. (2009) [66]	T2DM patients (n = 109)	1 g/day *Cinnamomum cassia* 3 months	HbA_1c_ decrease
Khan, R. (2010) [67]	T2DM patients (n = 14)	1.5 g/day cinnamon 1 month	FPG decrease
Akilen, R. (2010) [68]	T2DM patients (n = 58)	2 g/day *Cinnamomum cassia* 3 months	FPG and HbA_1c_ decrease
Markey, O. (2011) [69]	Healthy young subjects (n = 9)	High fat meal with 3 g *Cinnamomum zeylanicum* 3 h	PPG no decrease
Vafa, M. (2012) [70]	T2DM patients (n = 44)	3 g/day *Cinnamomum zeylanicum* 8 weeks	FPG and HbA_1c_ decrease
Sharma, P. (2012) [71]	Newly diagnosed T2DM patients (n = 150)	3 g/day and 6 g/day cinnamon 3 months	FPG and HbA_1c_ decrease
Lu, T. (2012) [72]	T2DM patients (n = 66)	120 mg/day and 360 mg/day *Cinnamomum aromaticum* 3 months	FPG and HbA_1c_ decrease
Hasanzade, F. (2013) [73]	T2DM patients (n = 70)	2 g/day *Cinnamomum cassia* 2 months	FPG and HbA_1c_ no change
Wickenberg, J. (2014) [74]	IGT patients (n = 21)	12 g/day *Cinnamomum cassia* 12 weeks	FPG, FPI and HbA_1c_ no change
Al-Yassiry, K. (2014) [75]	T2DM patients (n = 40)	1.5 g/day crude grind cinnamon 3 months	FPG, RBS and HbA_1c_ decrease
Beejmohun, V. (2014) [76]	Healthy volunteers (n = 18)	1 g Ceylon cinnamon hydro-alcoholic extract 30 min before the test meal	PPG and glucose AUC decrease
Bernardo, M.A. (2015) [77]	Nondiabetic subjects (n = 30)	6 g/100 mL of *Cinnamomum burmannii* tea 2 h	Maximum PPG concentration and variation in maximum glucose concentration decrease
Mirfeizi, M. (2016) [78]	T2DM patients (n = 105)	1 g/day cinnamon 3 months	FPG, 2h-PPG, FPI, HbA_1c_ and HOMA-IR decrease
Anderson, R.A. (2016) [79]	Patients with FPG > 100 mg/dL or 2h-PPG > 140 mg/dL (n = 137)	500 mg/day *Cinnamomum cassia* 2 months	FPG, 2h-PPG, FPI, and HOMA-IR decrease
Talaei, B. (2017) [80]	T2DM patients (n = 44)	3 g/day cinnamon 8 weeks	FPG, FPI, HbA_1c_, and HOMA-IR no change
Gupta Jain, S. (2017) [81]	Metabolic syndrome patients (n = 116)	3 g/day cinnamon 16 weeks	FPG, PPG, and HbA_1c_ decrease
Hajimonfarednejad, M. (2018) [82]	Women with PCOS (n = 66)	1.5 g/day cinnamon 12 weeks	FPI and HOMA-IR decrease
Zare, R. (2019) [83]	T2DM patients (n = 140)	1 g/day cinnamon bark powder 3 months	FPG, 2h-PPG, HbA_1c_, FPI, and HOMA-IR decrease
Kizilaslan, N. (2019) [84]	Healthy subjects (n = 41)	1 g/day, 3 g/day and 6 g/day cinnamon 40 days	FPG decrease at the dose of 6 g/day; 2h-PPG decrease at the dose of 1 g/day, 3 g/day and 6 g/day
Lira Neto, J.C.G. (2022) [85]	T2DM patients (n = 160)	3 g/day *Cinnamomum verum* 3 months	FPG, HbA_1c_ and HOMA-IR decrease
Rachid, A.P. (2022) [86]	T2DM patients (n = 36)	6 g/100 mL of aqueous cinnamon extract 2 h	iAUC glucose, maximum glucose concentration and glucose concentration variation no change
Liu, Y. (2015) [87]	Obese or overweight prediabetic subjects (n = 62)	1.2 g/day *Cinnamomum cassia*, chromium and carnosine 4 months	FPG decrease
Whitfield, P. (2016) [88]	T2DM patients (n = 12)	53.5 g/day of kanuba honey formulated with 4.5 g food grade cinnamon, 200 μg chromium picolinate and 120 mg magnesium citrate 40 days	FPG, FPI and HbA_1c_ no change

iAUC: incremental area under the curve; FPG: fasting plasma glucose; FPI: fasting plasma insulin; HbA_1c_: glycated hemoglobin; HOMA-IR: homeostatic model assessment insulin resistance; IGT: impaired glucose tolerance; ISIcomp: composite index of insulin sensitivity; OGIS: oral glucose insulin sensitivity; PCOS: polycystic ovarian syndrome; 2h-PPG: 2 h post-prandial plasma glucose; PPI: post-prandial plasma insulin; QUICKI: quantitative insulin sensitivity check index; RBS: random blood sugar; T1DM: type 1 diabetes mellitus; T2DM: type 2 diabetes mellitus.

**Table 6:** Overview of clinical trials on hypoglycemic activity of *Ilex paraguariensis*.

First Author and Year	Participants	Therapy and Duration	Findings
Arcari, D.P. (2011) [110]	Normolipidemic subjects (n = 42) Hyperlipidemic patients (n = 18)	200 mL maté tea (12.5 mg/mL) per day 2 months	FPG no decrease
Klein, G.A. (2011) [111]	T2DM patients (n = 29) Prediabetic patients (n = 29)	19.8 g yerba mate leaves to 990 of boiling water per day 60 days	FPG and HbA_1c_ decrease in T2DM patients
Kim, H.J. (2012) [112]	Overweight patients (n = 46)	334 mg/day green mate powder extract 6 weeks	FPG no decrease
Boaventura, B.C.B. (2013) [113]	T2DM patients (n = 11) Prediabetic patients (n = 11)	19.8 g yerba mate leaves to 990 of boiling water per day 60 days	FPG and HbA_1c_ decrease in T2DM patients HbA_1c_ decrease in prediabetics
Jung, J. (2016) [114]	Obese women (n = 33)	3 g/day maté extract 6 weeks	FPG no decrease
Derosa, G. (2020) [115]	IFG, IGT patients (n = 137)	1 tablet/day *Ilex paraguariensis* 500 mg + white mulberry 50 mg + chromium picolinate 100 μg 3 months	FPG and HOMA index decrease M value increase
Derosa, G. (2021) [116]	IFG, IGT patients (n = 143)	1 tablet/day *Ilex paraguariensis* 1000 mg + white mulberry 50 mg + chromium picolinate 100 μg 3 months	FPG, PPG, HbA_1c_ and HOMA index decrease M value increase

FPG: fasting plasma glucose; IFG: impaired fasting glucose; IGT: impaired glucose tolerance; HbA_1c_: glycated hemoglobin; HOMA index: homeostatic model assessment index; PPG: post-prandial plasma glucose; T2DM: type 2 diabetes mellitus.

**Table 7:** Recapitulation of clinical trials on *Momordica charantia* glucose-lowering activity.

First Author and Year	Participants	Therapy and Duration	Findings
Baldwa, V.S. (1977) [127]	T2DM patients (n = 9)	Vegetable insulin 10 units for FPG < 180 mg/dL 20 units for FPG > 180 and <250 mg/dL 30 units for FPG ≥ 250 mg/dL	Blood glucose levels decrease
Leatherdale, B.A. (1981) [128]	T2DM patients (n = 9)	50 mL water-soluble extract of bitter melon fruit 0.23 kg/day fried bitter melon fruit 8–11 weeks	PPG and glucose iAUC decrease (water-soluble extract of bitter melon fruit) Glucose iAUC decrease (fried bitter melon fruit)
Khann, P. (1981) [129]	T2DM patients (n = 19)	Polypeptide-p 10 units for FPG < 180 mg/dL 20 units for FPG > 180 and <250 mg/dL 30 units for FPG ≥ 250 mg/dL	Blood glucose levels decrease
Akhtar, M.S. (1982) [130]	T2DM patients (n = 8)	50 mg/kg *Momordica charantia* fruit 7 days	PPG decrease
Welihinda, J. (1986) [131]	T2DM patients (n = 18)	100 mL *Momordica charantia* juice 3 h	PPG and total area under *Momordica charantia* glucose tolerance curves decrease
John, A.J. (2003) [132]	T2DM patients (n = 50)	6 g/day *Momordica charantia* dried powdered fresh whole fruit 4 weeks	FPG and PPG no decrease
Tongia, A. (2004) [133]	T2DM patients, non-insulin-dependent (n = 15)	(1) 1 g/day metformin; 10 mg/day glibenclamide; 1 g/day metformin and 10 mg/day glibenclamide mixture 7 days (2) 400 mg/day *Momordica charantia* fruit extract with 500 mg/day metformin or 5 mg/day glibenclamide or 500 mg/day metformin and 5 mg/day glibenclamide mixture 7 days	FPG and HbA_1c_ no decrease
Dans, A.M. (2007) [134]	T2DM patients (n = 40)	3 g/day *Momordica charantia* capsules 3 months	PPG and PPI decrease
Inayat-ur-Rahman, S.A. (2009) [135]	T2DM patients, non-insulin-dependent (n = 50)	55 mL/day *Momordica charantia* juice or 4 mg/day rosiglitazone 6 months	Serum glucose no decrease
Kasbia, G.S. (2009) [136]	Overweight subjects (n = 5)	50 mg/kg or 100 mg/kg freeze dried *Momordica charantia* juice 3 h	FPG, FPI, PPG, and PPI no changes
Lim, S.T. (2010) [137]	T2DM patients (n = 40)	60 mg/kg 80 mg/kg or 100 mg/kg dried *Momordica charantia* leaves 8–11 weeks	PPI increase PPG decrease
Fuangchan, A. (2011) [138]	T2DM patients (n = 120)	500 mg/day, 1 g/day and 2 g/day *Momordica charantia* or 1 g/day metformin 4 weeks	FPG and 2h-PPG no changes
Tsai, C.H. (2012) [139]	Metabolic syndrome subjects (n = 42)	4.8 g/day bitter gourd powder 3 months	logHOMA decrease QUICKI and McAuley increase
Inayat, U.; Rahman, R.U. (2015) [140]	T2DM patients (n = 95)	2 g/day and 4 g/day bitter melon or 5 mg/day glibenclamide 10 weeks	FPG and HbA_1c_ decrease to a greater extent with glibenclamide 2h-PPG decrease with glibenclamide
Krawinkel, M.B. (2018) [141]	Prediabetic patients (n = 52)	2.5 g/day dry bitter gourd 2 months	FPG decrease
Cortez-Navarrete, M. (2018) [142]	T2DM patients (n = 25)	2 g/day *Momordica charantia* fruit powder 3 months	HbA_1c_, 2h-PPG, and AUC glucose decrease AUC insulin, insulinogenic, and Stumvoll index increase
Kim, S.K. (2020) [143]	T2DM patients (n = 90)	2.38 g/day bitter melon extract 12 weeks	FPG and HOMA-IR decrease
Kochhar, A. (2005) [144]	T2DM patients, non-insulin-dependent (n = 60)	(1) 1 g/day *Momordica charantia* fruit powder + fenugreek seeds + jamun seeds mixture 1.5 months (2) 2 g/day *Momordica charantia* fruit powder + fenugreek seeds + jamun seeds mixture 1.5 months	FPG and PPG decrease with both doses Reduction in percentage of subjects on oral hypoglycemic drug

iAUC: incremental area under the curve; FPG: fasting plasma glucose; FPI: fasting plasma insulin; HbA_1c_: glycated hemoglobin; HOMA-IR: homeostatic model assessment insulin resistance; 2h-PPG: 2 h post-prandial plasma glucose; PPI: post-prandial plasma insulin; QUICKI: quantitative insulin sensitivity check index; T2DM: type 2 diabetes mellitus.

**Table 8:** Overview of the clinical trial on *Morus* glucose-lowering effects.

First Author and Year	Participants	Therapy and Duration	Findings
Kimura, T. (2007) [150]	Healthy subjects (n = 24)	(1) *Morus alba* powder leaves enriched with 0.4, 0.8, or 1.2 g DNJ 3 h (2) *Morus alba* powder leaves enriched with 3.6 g/day DNJ 38 days	(1) PPG and PPI decrease (2) FPG no decrease
Mudra, M. (2007) [152]	T2DM patients (n = 10) Controls (n = 10)	*Morus alba* leaf extract 1 g/day 1 week	PPG decrease
Asai, A. (2011) [153]	(1) Dysglycemic patients (n = 12) (2) Dysglycemic patients (n = 76)	(1) *Morus alba* leaf extract enriched with 3, 6, or 9 mg DNJ 2 h (2) *Morus alba* leaf extract enriched with 18 g/day DNJ 12 weeks	(1) PPG and PPI decrease (2) FPG, FPI, HbA_1c_, and GA no decrease 1,5AG decrease
Nakamura, S. (2011) [154]	T2DM patients (n = 10) Healthy subjects (n = 10)	3.3 g *Morus alba* leaf extract 2 h	PPG and PPI decrease
Chung, H.I. (2013) [155]	Healthy subjects (n = 50)	1.25, 2.5, or 5 g *Morus alba* leaf aqueous extract 3 h	PPG decrease
Kim, J.Y. (2015) [156]	IFG patients (n = 38)	5 g/day *Morus alba* leaf aqueous extract 4 weeks	PPG, PPI, insulin AUC, and C-peptide decrease
Banu, S. (2015) [157]	T2DM patients (n = 48)	70 mL *Morus alba* leaf tea 1.30 h	FPG and PPG decrease
Sukriket, P. (2016) [158]	Non-diabetic subjects (n = 14)	2 g *Morus alba* leaf tea powder 2.30 h	PPG decrease
Riche, D.M. (2017) [159]	T2DM patients (n = 17)	3 g/day *Morus alba* leaf extract 3 months	Post-prandial SMBG and HbA_1c_ decrease
Thaipitakwong, T. (2020) [160]	(1) Healthy subjects (n = 85) (2) Obese (n = 54)	(1) *Morus alba* leaf powder with DNJ 6, 12 and 18 mg 3 h (2) *Morus alba* leaf powder with DNJ 36 mg/day 12 weeks	(1) PPG decrease (2) FPG and HbA_1c_ decrease
Thondre, P.S. (2021) [161]	Healthy subjects (n = 38)	250 mg *Morus alba* leaf aqueous extract 2 h	PPG, PPI, glucose, and insulin iAUC decrease
Momeni, H. (2021) [162]	T2DM patients (n = 100)	9 mL/day 4% hydro-alcoholic extract of *Morus nigra* leaves 3 months	FPG and HbA_1c_ decrease
Kim, H.J. (2012) [26]	IGT and mild-T2DM patients (n = 62)	6 g/day white mulberry leaf water extract powder, Korean red ginseng powder, and banaba leaf water extract powder 24 weeks	OGTT AUC glucose decrease FPG, FPI, HOMA-IR, and HbA_1c_ no decrease
Hu, M. (2014) [163]	Dyslipidemia patients (n = 40)	8 capsules/day each containing *Crataegus pinnatifida* 129 mg, *Alisma orientalis* 86 mg, *Stigma maydis* 86 mg, *Ganoderma lucidum* 43 mg, *Polygonum multiflorum* 43 mg and *Morus alba* 43 mg 12 weeks	HbA_1c_ decrease
Trimarco, V. (2015) [164]	Hypercholesterolemia patients (n = 23)	Combination A: Policosanol (10 mg), red yeast rice (200 mg; 3 mg monacolin K), berberine (500 mg), astaxanthin (0.5 mg), folic acid (200 μg) and coenzyme Q10 (2 mg) Combination B: Berberine (531.25 mg), red yeast rice powder (220 mg; 3.3 mg monacolin K), and leaf extract of *Morus alba* (200 mg) 8 weeks	FPG FPI, HbA_1c_, and HOMA index decrease with combination B
Derosa, G. (2020) [115]	IFG, IGT patients (n = 137)	1 tablet/day white mulberry 50 mg + *Ilex paraguariensis* 500 mg + chromium picolinate 100 μg 3 months	FPG and HOMA index decrease M value increase
Derosa, G. (2021) [116]	IFG, IGT patients (n = 143)	1 tablet/day white mulberry 50 mg + *Ilex paraguariensis* 1000 mg + chromium picolinate 100 μg 3 months	FPG, PPG, HbA_1c_, and HOMA index decrease M value increase

1,5AG: 1,5-anhydroglucitol; iAUC: incremental area under the curve; FPG: fasting plasma glucose; FPI: fasting plasma insulin; GA: glycated albumin; HbA_1c_: glycated hemoglobin; HOMA-IR: homeostatic model assessment of insulin resistance; IFG: impaired fasting glucose; IGT: impaired glucose tolerance; OGTT: oral glucose tolerance test; PPG: post-prandial plasma glucose; PPI: post-prandial plasma insulin; SMBG: self-monitoring blood glucose; T2DM: type 2 diabetes mellitus.

**Table 9:** Recapitulation of research on hypoglycemic effects of *Olea europaea* in humans.

First Author and Year	Participants	Therapy and Duration	Findings
Wainstein, J. (2012) [171]	T2DM patients (n = 79)	500 mg/day OLE 14 weeks	HbA_1c_ and FPI decrease
De Bock, M. (2013) [172]	Overweight subjects (n = 46)	4 capsules/day OLE (51.1 mg oleuropein and 9.7 mg hydroxytyrosol) 12 weeks	Insulin sensitivity and pancreatic β-cell function improvement PPG, PPI, glucose AUC, and insulin AUC decrease
Santangelo, C. (2016) [173]	Overweight T2DM patients (n = 11)	(1) 25 mL/day ROO 4 weeks (2) 25 mL/day HP-EVOO 4 weeks	FPG and HbA_1c_ decrease with HP-EVOO
Araki, R. (2019) [174]	Subjects with prediabetes (n = 57)	1.5 g/day or 15 g/day OLT 12 weeks	FPG decrease
Derosa, G. (2022) [175]	IFG patients (n = 148)	70 mL/day infusion of olive leaves and marigold (Olife^®^) 3 months	FPG, PPG, and HOMA index decrease
Said, O. (2008) [176]	(1) T2DM patients (n = 16) (2) T2DM patients (n = 22)	3 tablets/day dry extract of *Olea europea* L. + *Juglans regia* L. + *Urtica dioica* L. + *Atriplex halimus* L. 4 weeks	(1) FPG decrease (2) HbA_1c_ decrease
Wong, R.H. (2014) [177]	Subjects with mildly elevated untreated BP (n = 37)	1 g/day OLE + 200 mg/day coffee bean extract + 300 mg/day beet powder 12 weeks	FPG, FPI, and HOMA index no change
Derosa, G. (2018) [178]	Hypercholesterolemic overweight subjects (n = 80)	25 mg/day olive fruit extract + 166 mg/day fermented red rice + 720 mg/day sterol esters and stanols + 230 mg/day curcumin 3 months	FPG no change
Hermans, M.P. (2020) [179]	Subjects with borderline hypertension or grade 1 hypertension (n = 663)	2 capsules/day Tensiofytol^®^ (167 mg *Olea europea* L. leaf dry extract + 53 mg *Olea europea* L. fruit dry extract) 2 months	FPG decrease
Chavenelle, V. (2021) [180]	Overweight subjects (n = 14)	(1) 2.5 g/day TOTUM-63 (extracts from olive leaf, bilberry, artichoke, Chrysanthellum, and black pepper) 4 weeks (2) 5 g/day TOTUM-63 4 weeks 2 weeks of wash-out between the two treatments	PPG, glucose peak (Cmax), PPI, insulin peak (Cmax), insulin AUC and insulin sensitivity index decrease with 5 g/day of TOTUM-63
Sirvent, P. (2022) [181]	Subjects with prediabetes or untreated T2DM (n = 51)	5 g/day TOTUM-63 6 months	FPG and 2h-PPG decrease

AUC: area under the curve; BP: blood pressure; FPG: fasting plasma glucose; FPI: fasting plasma insulin; HbA_1c_: glycated hemoglobin; HOMA: homeostatic model assessment; HP-EVOO: high polyphenol extra-virgin olive oil; IFG: impaired fasting glucose; OLE: olive leaf extract; OLT: olive leaf tea; 2h-PPG: 2 h post-prandial plasma glucose; PPI: post-prandial plasma insulin; ROO: refined olive oil; T2DM: type 2 diabetes mellitus.

**Table 10:** Overview of human studies on glucose-lowering effects of alpha-lipoic acid.

First Author and Year	Participants	Therapy and Duration	Findings
Morcos, M. (2001) [184]	T1DM and T2DM patients (n = 84)	600 mg/day ALA 18 months	HbA_1c_ not altered
Kamenova, P. (2006) [185]	T2DM patients (n = 12)	1200 mg/day ALA 4 weeks	Glucose metabolized (M value) and ISI increase
Lukaszuk, J.M. (2009) [186]	T2DM patients (n = 12)	600 mg/day R-ALA 91 days	HbA_1c_ decreased in 2 subjects
Ansar, H. (2011) [187]	T2DM patients (n = 57)	300 mg/day ALA 8 weeks	FPG, PPG, and HOMA index decrease Glutathione peroxidase increase
Koh, E.H. (2011) [188]	Obese subjects with hypertension, T2DM or hypercholesterolemia (n = 360)	1200 or 1800 mg/day ALA 20 weeks	HbA_1c_ decrease at the dose of 1800 mg/day
Zhang, Y. (2011) [189]	Obese with IGT subjects (n = 22)	600 mg/day ALA 2 weeks	2h-PPG decrease M value and ISI increase
Porasuphatana, S. (2012) [190]	T2DM patients (n = 38)	300, 600, 900 and 1200 mg/day ALA 6 months	FPG and HbA_1c_ decrease dose-dependent
Udupa, A.S. (2012) [191]	T2DM patients (n = 104)	(1) 300 mg/day ALA soft gelatin capsules (2) EPA 180 mg + DHA 120 mg 6 soft gelatin capsules/day (3) 400 mg/day vitamin E soft gelatin capsules 3 months	HbA_1c_ decrease by ALA, omega-3 and vitamin E
Manning, P.J. (2013) [192]	Patients with metabolic syndrome (n = 160)	(1) 600 mg/day ALA (2) 100 IU/day vitamin E (3) 600 mg/day ALA + 100 IU/day vitamin E 12 months	FPG, insulin, and HOMA-IR not altered
Zhao, L. (2014) [193]	Aged T2DM patients with acute cerebral infarction (n = 90)	600 mg/day ALA or 3 g/day vitamin C 3 weeks	FPG, 2h-PPG, HbA_1c_, and HOMA-IR decrease HOMA-B increase
Okanović, A. (2015) [194]	Obese T2DM patients with signs of peripheral polyneuropathia (n = 60)	600 mg/day ALA 20 weeks	FPG, decrease
Scaramuzza, A. (2015) [195]	Adolescent with T1DM (n = 71)	400 mg/day ALA + antioxidant diet 3 months	Daily insulin requirement and percentage of bolus dose decrease
Surapaneni, K. (2018) [196]	T2DM patients with chronic periodontal disease (n = 40)	1800 mg/day ALA 3 months	HbA_1c_ decrease
Gosselin, L.E. (2019) [197]	Prediabetic and dyslipidemic patients (n = 12)	600 mg/day ALA 2 months separated by wash-out period of 1 month	FPI and HOMA-IR decrease
Derosa, G. (2020) [198]	Euglycemic and dysglycemic patients (n = 333)	400, 600, 800, and 1200 mg/day ALA 4 years	FPG decrease at the dose of 800 and 1200 mg/day ALA Some dysglycemic patients returned to euglycemic condition
De Oliveira, A.M. (2011) [199]	T2DM patients (n = 102)	(1) 600 mg/day ALA (2) 800 mg/day α-tocopherol (3) 600 mg/day ALA + 800 mg/day α-tocopherol 4 months	FPG, insulin and HOMA index not altered
Derosa, G. (2016) [200]	T2DM patients (n = 105)	600 mg ALA + 165 mg L-carnosine + 7.5 mg zinc + vitamins B complex daily 3 months	FPG, PPG, HbA_1c_, and HOMA-IR decrease Superoxide dismutase and glutathione peroxidase increase Malondialdehyde decrease
Cicero, A.F.G. (2017) [27]	IFG patients (n = 40)	220 mg/day ALA + 310 mg/day berberine + 500 mg/day *Lagerstroemia speciosa* + 250 mg/day curcumin + 2.6 μg/day chromium picolinate + 0.30 mg/day folic acid 8 weeks	FPG, FPI and HOMA index decrease
Karkabounas, S. (2018) [201]	Obese T2DM patients (n = 82)	7 mg ALA/kg body weight + 6 mg carnosine/kg body weight + 1 mg thiamine/kg body weight, 3 times/day 8 weeks	FPG, and HbA_1c_ decrease Insulin and HOMA-B increase QUICKI decrease
Derosa, G. (2022) [202]	T2DM patients with erectile dysfunction (n = 123)	400 mg ALA + 200 mg *Vitis vinifera* L. + 80 mg *Ginkgo biloba* daily 200 mg/day Avanafil 3 months	FPG, and HOMA-IR decrease

ALA: alpha-lipoic acid; DHA: docosahexaenoic acid; EPA: eicosapentaenoic acid; FPG: fasting plasma glucose; FPI: fasting plasma insulin; HbA_1c_: glycated hemoglobin; HOMA-B: homeostatic model assessment of β cell function; HOMA-IR: homeostatic model assessment of insulin resistance; IGT: impaired glucose tolerance; ISI: insulin sensitivity index; 2h-PPG: 2 h post-prandial plasma glucose; QUICKI: quantitative insulin sensitivity check index; T1DM: type 1 diabetes mellitus; T2DM: type 2 diabetes mellitus.

**Table 11:** Recapitulation of clinical studies on hypoglycemic effects of omega-3.

First Author and Year	Participants	Therapy and Duration	Findings
Glauber, H. (1988) [213]	T2DM non-insulin dependent patients (n = 6)	5.5 g/day *n*-3 PUFAs 1 months	FPG and HbA_1c_ increase FPI slightly not significant decrease
Friday, K.E. (1989) [214]	T2DM non-insulin dependent patients (n = 8)	8.0 g/day *n*-3 PUFAs 8 weeks	FPG increase FPI and HbA_1c_ not altered
Borkman, M. (1989) [215]	T2DM non-insulin dependent patients (n = 10)	3.0 g/day *n*-3 PUFAs 3 weeks	FPG increase FPI and C-peptide not altered
Rillaerts, E.G. (1989) [216]	Type 1 diabetes mellitus patients (n = 12)	2.7 g/day *n*-3 PUFAs 10 weeks	FPG and HbA_1c_ not altered
Hendra, T.J. (1990) [217]	T2DM non-insulin dependent patients (n = 80)	3.0 g/day *n*-3 PUFAs 6 weeks	FPG increase HbA_1c_ not altered
Pelikánová, T. (1993) [218]	T2DM non-insulin dependent, obesity patients (n = 20)	3.1 g/day *n*-3 PUFAs 3 weeks	FPG, PPG and HbA_1c_ not altered
Morgan, W.A. (1995) [219]	Patients with T2DM non-insulin dependent and hyperlipidemia (n = 40)	(1) 5.0 g/day *n*-3 PUFAs (2) 10.0 g/day *n*-3 PUFAs 12 weeks	FPG and HbA_1c_ not altered
Goh, Y.K. (1997) [220]	T2DM non-insulin dependent patients assigned to a higher or lower ratio of dietary polyunsaturated to saturated fatty acid (P/S) (n = 28)	(1) 35 mg Oleic acid per kg body weight per day 3 months (2) 35 mg linseed oil or fish oli per kg body weight per day 3 months	FPG, insulin, glucagon, C-peptide not altered
Sirtori, C.R. (1998) [221]	T2DM non-insulin dependent patients (n = 414)	(1) 2.6 g/day *n*-3 PUFAs 2 months (2) 1.7 g/day *n*-3 PUFAs 10 months	FPG, FPI and HbA_1c_ not altered
Kabir, M. (2007) [222]	T2DM patients (n = 26)	1.8 g/day *n*-3 PUFAs 2 months	FPG, FPI, HbA_1c_, HOMA-B and HOMA-IS not altered
Derosa, G. (2009) [223]	Patients with combined dyslipidemia (n = 333)	3.0 g/day *n*-3 PUFAs 6 months	FPG, FPI, and HOMA-IR not altered
Wong, C.Y. (2010) [224]	T2DM patients (n = 97)	2.7 g/day *n*-3 PUFAs 12 weeks	FPG and HbA_1c_ not altered
Fakhrzadeh, H. (2010) [225]	Elderly patients (n = 124)	300 mg/day *n*-3 PUFAs 6 months	FPG, FPI and HOMA-IR not altered
Cicero, A.F. (2010) [226]	Patients with hypertriglyceridemia and untreated normal-high blood pressure (n = 111)	2.0 g/day *n*-3 PUFAs 12 months	FPG not altered except an increase at 3 months
Derosa, G. (2011) [227] Derosa, G. (2012) [228]	Patients with combined dyslipidemia (n = 167)	3.0 g/day *n*-3 PUFAs 6 months	FPG decrease FPI and HOMA-IR not altered M value and TGR increase
ORIGIN Trial Investigators (2012) [229]	IFG, IGT patients (n = 12.536)	1.0 g/day *n*-3 PUFAs Follow-up 6.2 years	FPG and HbA_1c_ not altered
Crochemore, I.C. (2012) [230]	Patients with T2DM and hypertension (n = 41)	(1) 900 mg/day *n*-3 PUFAs (2) 540 mg/day *n*-3 PUFAs 1 months	(1) QUICKI decrease in 85.7% of subjects (2) FPG, HbA_1c_ and HOMA-IR decrease in 42.9%, 35.7% and 35.7% of subjects
Mansoori, A. (2015) [231]	T2DM patients (n = 72)	1.85 g/day *n*-3 PUFAs 8 weeks	FPG, FPI and HOMA-IR not altered
Derosa, G. (2016) [232]	IFG, IGT, overweight/obesity patients (n = 281)	3.0 g/day *n*-3 PUFAs 18 months	FPG, FPI, and HOMA-IR decrease
Sawada, T. (2016) [233]	IGM patients with CAD (n = 107)	1800 mg/day EPA 6 months	FPG, HbA_1c_, and HOMA-IR not altered Glucose AUC and incremental glucose peak decrease Immune reactive insulin AUC increase Immune reactive insulin AUC/glucose AUC ratio increase
Jacobo-Cejudo, M.G. (2017) [234]	T2DM patients (n = 54)	520 mg/day *n*-3 PUFAs 6 months	FPG and HbA_1c_ decrease FPI and HOMA-IR increase
Thota, R.N. (2019) [235]	IFG, IGT patients (n = 64)	1.2 g/day *n*-3 PUFAs + 180 mg/day curcumin 12 weeks	FPG, HbA_1c_, FPI, HOMA-IR, and HOMA-IS not altered

CAD: coronary artery disease; EPA: eicosapentaenoic acid; FPG: fasting plasma glucose; FPI: fasting plasma insulin; HbA_1c_: glycated hemoglobin; HOMA-B: homeostatic model assessment of β cell function; HOMA-IR: homeostatic model assessment of insulin resistance; HOMA-IS: homeostatic model assessment of insulin sensitivity; IFG: impaired fasting glucose; IGM: impaired glucose metabolism; IGT: impaired glucose tolerance; *n*-3 PUFAs: omega-3 polyunsaturated fatty acids; QUICKI: quantitative insulin sensitivity check index; T2DM: type 2 diabetes mellitus; TGR: total glucose requirement.

**Table 12:** Overview of human studies on hypoglycemic effects of essential amino acids.

First Author and Year	Participants	Therapy and Duration	Findings
Hedo J.A., (1977) [239]	Healthy subjects (n = 21)	10 g L-tryptophan 180 min	Blood sugar levels highest increase at 180 min Insulin highest increase at 140 min
Collene A.L., (2005) [240]	Healthy subjects (n = 43)	(1) Control (C) (2) C + phenylalanine 3.5 g + leucine 3.5 g (AA) (3) C + *Salacia oblonga* extract 1 g (S) (4) C + *Salacia oblonga* extract 1 g + phenylalanine 3.5 g + leucine 3.5 g (SAA) Separate days	Glucose AUC 0–120 min decrease for S and SAA Glucose AUC 0–180 min decrease for SAA Insulin AUC 0–120 min and 0–180 min decrease for S and SAA
Monti L.D., (2012; 2018) [241,242]	IGT and metabolic syndrome subjects (n = 144)	L-arginine 6.4 g/day 18 months	Improvement of insulin levels at 120 min after OGTT, proinsulin/c-peptide ratio, IGI/HOMA-IR, and cumulative probability to return to euglycemia. After 12-month extended follow-up period: improvement of insulin levels at 120 min after OGTT, proinsulin/c-peptide ratio, IGI/HOMA-IR and cumulative probability to return to euglycemia; 2h-PPG, HbA_1c_ and cumulative incidence of diabetes decrease. After a 90-month extended follow-up period: cumulative incidence of diabetes decrease; improvement of proinsulin/c-peptide ratio and IGI/HOMA-IR
Steinert, R.E. (2014) [243]	Healthy normal-weight men (n = 10)	90-min intraduodenal L-tryptophan infusion at 0.075 or 0.15 kcal/min 3 occasions	Insulin increase 75 min after infusion with L-tryptophan 0.075 kcal/min Blood glucose not altered with both doses
Steinert, R.E. (2015) [244]	Lean men (n = 12)	90-min intraduodenal leucine infusion at 0.15 or 0.45 kcal/min 3 occasions	Blood glucose decrease with leucine 0.45 Insulin increase with both doses
Ullrich, S.S. (2016) [245]	Healthy subjects (n = 12)	(1) Intragastric leucine infusion 5 g or 10 g (2) Intragastric isoleucine infusion 5 g or 10 g 3 separate visits	Glucose AUC decrease with leucine 10 g and isoleucine 10 g Insulin and C-peptide AUCs increased with leucine 10 g
Ullrich, S.S. (2017) [246]	Healthy volunteers (n = 12)	Intragastric L-lysine infusion 5 g or 10 g Mixed-nutrient drink after 15 min 3 occasions	Blood glucose, insulin, blood glucose, and insulin AUCs not altered Blood glucose and insulin decrease in response to mixed-nutrient drink
Randolph, A.C. (2020) [247]	Healthy older adults (n = 42)	15 g/day supplement contained 40% L-leucine, 16.7% L-lysine, 11% L-valine, 10.7% L-isoleucine, 9.3% L-threonine, 6.7% L-phenylalanine, 3.3% L-methionine, 1.7% histidine, 0.7% L-tryptophan alone or in association with aerobic exercise 22 weeks	Insulin sensitivity not altered
Amin, A. (2021) [248]	Healthy subjects (n = 11)	10 g L-phenylalanine Test meal 70 min after treatment 120 min	PPG AUC decrease Insulin increase prior meal intake Insulin AUC increase after meal intake Glucagon increase before and after meal intake GIP increase after treatment
Elovaris, R.A. (2021) [249]	Healthy subjects (n = 15)	10 g leucine, isoleucine and valine Mixed-nutrient drink 30 min after treatment 4 separate occasions	Peak glucose decrease with leucine and isoleucine Glucose AUC_15–120min_ reduced with isoleucine
Elovaris, R.A. (2021) [250]	T2DM patients (n = 14)	10 g leucine and isoleucine Mixed-nutrient drink 30 min after treatment 3 separate visits	Glucose AUC and peak glucose levels not altered Insulin AUC increase before and after mixed-nutrient drink Peak insulin levels increase with leucine Glucagon AUC increase before and after mixed-nutrient drink with isoleucine
Alqudah, A. (2021) [236]	T2DM patients (n = 124) Healthy (n = 67)	Amino acids profile and correlation with glycemic parameters	Increase in leucine, lysine, phenylalanine, and tryptophan Positive correlation with FPG and HbA_1c_
Hajishafiee, M. (2021) [251]	T2DM patients (n = 12)	3 g or 1.5 g tryptophan Mixed-nutrient drink 30 min after treatment 3 separate occasions	FPG not altered C-peptide increase C-peptide AUC increase with 1.5 g tryptophan PPG decrease with 3 g tryptophan
Matsuda, T. (2022) [252]	T2DM elderly patients (n = 36)	8 g/day BCAA (4 g leucine, 2 g valine, 2 g isoleucine) 24 weeks	FPG, FPI, HbA_1c_, and HOMA-IR not altered

AUC: area under the curve; BCAA: branched-chain amino acid; FPG: fasting plasma glucose; FPI: fasting plasma insulin; GIP: glucose-dependent insulinotropic peptide; HbA_1c_: glycated hemoglobin; HOMA-IR: homeostatic model assessment of insulin resistance; IGI: insulinogenic index; IGT: impaired glucose tolerance; OGTT: oral glucose tolerance test; 2h-PPG: 2 h post-prandial plasma glucose; T2DM: type 2 diabetes mellitus.

## Abbreviations

ACC: acetyl-CoA carboxylase; 1,5AG: 1,5-anhydroglucitol; AGEs: advance glycation end-products; AKT: serine/threonine kinase; ALA: alpha-linolenic acid; AMPK: adenosine monophosphate-activated protein kinase; ATP: adenosine triphosphate; AUC: area under the curve; iAUC: incremental area under the curve; BCAA: branched-chain amino acid; BE: banaba extract; BMI: body mass index; BP: blood pressure; CAD: coronary artery disease; CCK: cholecystokinin; DHA: docosahexaenoic acid; DHLA: dihydro-lipoic acid; DNJ: 1-deoxynojirimycin; EPA: eicosapentaenoic acid; FPG: fasting plasma glucose; FPI: fasting plasma insulin; GA: glycated albumin; G6Pase: glucose-6-phosphatase; GIP: glucose-dependent insulinotropic peptide; GLP-1; glucagon-like peptide-1; GLUT: glucose transporter; GS: *Gymnema sylvestre*; HbA_1c_: glycosylated hemoglobin; HOMA-B: homeostatic model assessment of β cell function; HOMA index: homeostasis model assessment index; HOMA-IR: homeostasis model assessment of insulin resistance; HOMA-IS: homeostatic model assessment of insulin sensitivity; HP-EVOO: high polyphenol extra-virgin olive oil; IFG: impaired fasting glucose; IGI: insulinogenic index; IGM: impaired glucose metabolism; IGT: impaired glucose tolerance; IR: insulin receptor; IRS-1: insulin receptor substrate type 1; IRS-2: insulin receptor substrate type 2; ISIcomp: composite index of insulin sensitivity; JSB: jinshuibao; KGM: konjac-glucomannan; MAPK: mitogen-activated protein kinase; MLAE: mulberry leaf aqueous extract; MLE: mulberry leaf extract; mTORC1: mammalian target of rapamycin complex 1; *n*-3 PUFAs: omega-3 polyunsaturated fatty acids; OGIS: oral glucose insulin sensitivity; OGTT: oral glucose tolerance test; OLE: olive leaf extract; OLT: olive leaf tea; OSA: om santal Adivasi; PCOS: polycystic ovarian syndrome; PEPCK: phosphoenolpyruvate carboxykinase; PI3K: phosphatidylinositol 3-kinase; PPAR-α/γ: *peroxisome proliferator-activated receptor*-α/γ; PPG: post-prandial plasma glucose; PPI: post-prandial insulin; PYY: peptide tyrosine–tyrosine; ROO: refined olive oil; RBS: random blood sugar; SGLT1: sodium glucose co-transporter 1; SLE: soybean leaf extract; SMBG: self-monitoring blood glucose; T1DM: type 1 diabetes mellitus; T2DM: type 2 diabetes mellitus; TGR: total glucose requirement.
